# Fluorescent Proteins as Genetically Encoded FRET Biosensors in Life Sciences

**DOI:** 10.3390/s151026281

**Published:** 2015-10-16

**Authors:** Bernhard Hochreiter, Alan Pardo Garcia, Johannes A. Schmid

**Affiliations:** Institute for Vascular Biology and Thrombosis Research, Medical University Vienna, Schwarzspanierstraße17, Vienna A-1090, Austria; E-Mails: bernhard.hochreiter@meduniwien.ac.at (B.H.); alanpardogarcia@gmail.com (A.P.G.)

**Keywords:** FRET, fluorescence, biosensors, imaging

## Abstract

Fluorescence- or Förster resonance energy transfer (FRET) is a measurable physical energy transfer phenomenon between appropriate chromophores, when they are in sufficient proximity, usually within 10 nm. This feature has made them incredibly useful tools for many biomedical studies on molecular interactions. Furthermore, this principle is increasingly exploited for the design of biosensors, where two chromophores are linked with a sensory domain controlling their distance and thus the degree of FRET. The versatility of these FRET-biosensors made it possible to assess a vast amount of biological variables in a fast and standardized manner, allowing not only high-throughput studies but also sub-cellular measurements of biological processes. In this review, we aim at giving an overview over the recent advances in genetically encoded, fluorescent-protein based FRET-biosensors, as these represent the largest and most vividly growing group of FRET-based sensors. For easy understanding, we are grouping them into four categories, depending on their molecular mechanism. These are based on: (a) cleavage; (b) conformational-change; (c) mechanical force and (d) changes in the micro-environment. We also address the many issues and considerations that come with the development of FRET-based biosensors, as well as the possibilities that are available to measure them.

## 1. Introduction

**Figure 1:**
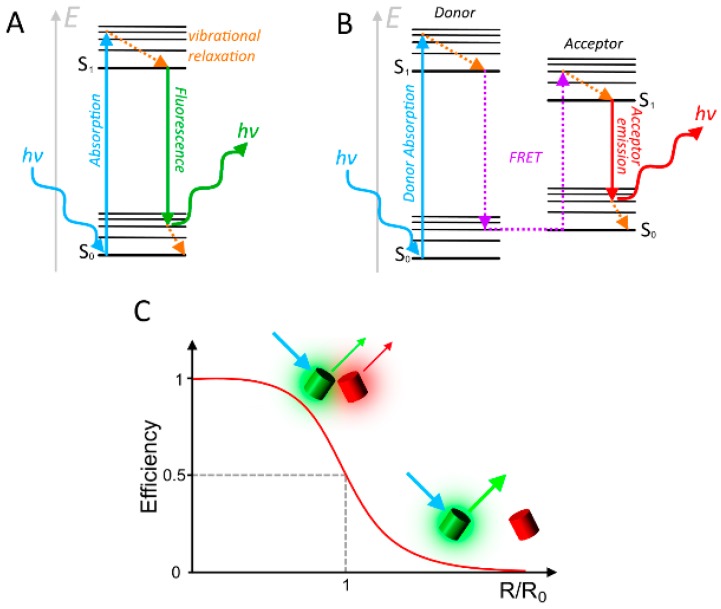
The basic principles of fluorescence and FRET. (**A**) Jablonski diagram, explaining the effect of fluorescence. Absorption of a photon by the fluorophore raises an electron to an excited energy state. Within this state, the electron drops to the ground level via radiationless vibrational relaxation. The excited state is very instable, leading to a relaxation back to the ground state within a few nanoseconds. The energy quantum of this difference is emitted via a photon of a specific wavelength, which is respectively longer than the absorbed wavelength, *i.e.*, contains less energy; (**B**) Jablonski Diagram explaining the effect of FRET. Here, the energy that is released from the relaxation of the donor is taken up by a suitable acceptor in close proximity, leading to the excitation of one of its electrons, and further to the emission of a photon by the acceptor rather than the donor; (**C**) Correlation between the FRET efficiency and the distance between the fluorophores. The Förster radius R_0_ is the distance, where 50% of energy is transferred via FRET.

Fluorescence is the phenomenon of light emission by a substance with appropriate chemical structure (also called fluorophore), after absorbing photons or electromagnetic radiation. The emitted light has usually a longer wavelength than the absorbed light due to some conversion of excitation energy into non-radiative processes. The phenomenon of fluorescence has been described already in the 16th century, while the term itself was coined in 1852 by G.G. Stokes [1]. Since then, the effect has been found in a wide array of inorganic and organic compounds and is often associated with conjugated double bonds providing appropriate energy states of the orbital electrons. Physically explained, the absorption of a photon by a fluorophore, leads to the excitation of an electron from its ground state S_0_, to an excited energy level S_1_. Depending on the specific wavelength, and therefore energy content, of the absorbed photon, the electron can rise to different vibrational levels within this excited state. Some energy of these levels is usually lost radiation-less via vibrational relaxation. The excited state is very instable, and the electron relaxes back to its ground state within nanoseconds, producing a photon with the corresponding energy quantum, respectively wavelength (Figure 1A). Due to the different vibrational levels at which an electron can arrive during both processes the absorption and emission does not occur only at a single precise wavelength, but rather within a wider spectrum of a specific range. This also accounts for the loss in energy, leading to an emission with lower energy content and therefore a higher wavelength, compared to the absorption wavelength. An exception to this rule occurs, when two or more photons act synchronously on a fluorophore (e.g., at high photon density resulting in two- or multi-photon excitation) so that the emitted light can have a shorter wavelength than the excitation light. An interesting variation to the phenomenon of fluorescence can occur, when two chromophores are in close proximity. In this case, part of the energy of the excited state of a fluorophore (termed as donor) can be transferred in a radiation-less manner by direct dipole-interaction to the second chromophore designated as acceptor (Figure 1B). This effect was first described by Theodor Förster in 1948, and hence is termed Förster Resonance Energy Transfer (FRET) [2]. Due to the common use of FRET with fluorophores, it is more commonly known as Fluorescence Resonance Energy Transfer nowadays. However, the second chromophore can-also be non-fluorescent, acting as a quencher of the donor fluorophore.

**Figure 2:**
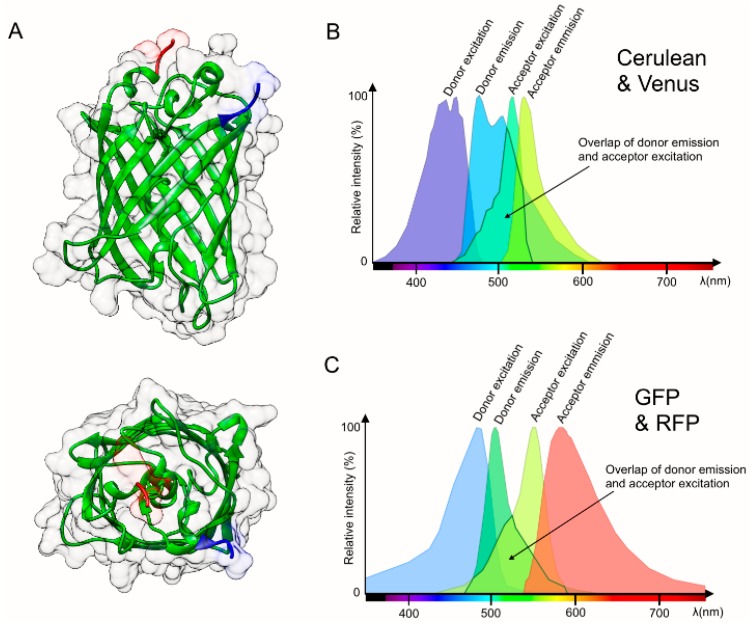
Structure and spectrum of fluorescent proteins. (**A**) The three-dimensional structure of Green fluorescent Protein (GFP) as it occurs naturally in the jellyfish species *Aequorea Victoria* [5]; (**B**,**C**) The spectrum of the fluorescent protein FRET-pair; (B) Cerulean (a cyan FP) and Venus (a yellow FP); and (C) GFP and RFP, also showing the overlap between donor emission and acceptor excitation, which is an important factor for the usability of a FRET-pair.

FRET has a specific array of requirements in order to occur. The first basic necessity for FRET is an overlap of the emission spectrum of the donor, and the excitation spectrum of the acceptor (Figure 2B,C), which is required for a sufficient energy coupling of the two compounds. It is usually assumed that an overlap of 30% or more is necessary to grant sufficient FRET for reliable detection [3]. The second requirement is a close proximity of donor and acceptor, ranging in a distance of 1–10 nm (see Table 1 for dynamic ranges of common protein FRET-pairs). In addition to a sufficient proximity of the chromophores, an appropriate relative orientation of the dipole vectors is necessary for a high FRET to occur. A high quantum yield of the donor, while not absolutely necessary can be very advantageous as it eases measurement and increases the yield of FRET signal donor [4]. Many biological processes such as protein interactions take place within the spatial range at which FRET occurs. Thus, FRET has found heavy use as a tool to monitor and visualize molecular interactions and furthermore has been applied as a spectroscopic ruler, because the distances that can be measured, are much shorter than the diffraction limit of conventional microscopy, and even super resolution microscopy (see Table 1).

The efficiency of FRET E is given by the ratio of the number of donor excitation events that result in FRET, to the total number of donor excitation events, and is directly related to the distance R between the fluorophores by the power of six (see Equation (1)):

(1) $$E=\frac{R_{0}^{6}}{R^{6}+R_{0}^{6}}$$

While *R* describes the actual distance between fluorophores, *R_0_* is called the Förster distance and describes the distance at which E is 0.5, or where exactly 50% of the donor excitation events lead to FRET (Figure 1C):

(2) $$R_{0}=0.2108{\left[\kappa^{2}\Phi_{0}n^{-4}J\right]}^{1/6}$$

*R_0_* is different for each FRET-pair, and is defined by the orientation factor κ, the quantum yield of donor fluorescence without acceptor Φ_0_, the refractive index of the intervening medium *n*, and the degree of spectral overlap between donor fluorescence spectrum (*F_D_*, integral normalised to 1) and acceptor absorption spectrum (scaled to its maximum molar extinction coefficient ε_*A*_), given by *J*:

(3) $$J=\overset{\infty }{\underset{0}{\int }}F_{D}\left(\lambda \right){\text{ε}}_{A}\left(\lambda \right)\lambda^{4}d\lambda$$

As *R_0_* of a given pair of chromophores represents a meaningful parameter to describe the capabilities of this pair to produce FRET under certain conditions, it is often used as one of the quality criteria to describe and select FRET-pairs. There are several other factors that have an impact on the actual effectivity of FRET and which were thoroughly reviewed recently by Shrestha *et al.* [4].

Because FRET efficiency and distance of donor and acceptor are relatable, FRET has found many applications in the last decades, often to prove the interaction or co-localization of two probes, which are bound to fluorophores. A particularly important discovery in relation to FRET was the description of Green Fluorescent Protein (GFP) in 1962 by Osamu Shimomura [6]. GFP is a naturally occurring fluorescent protein in the jellyfish species *Aequorea victoria*. The elucidation of the DNA sequence coding for GFP, and the expression of GFP outside of *Aequorea victoria* by Chalfie and Prasher in the early 90s [7,8] boosted its application in biological research, because it allowed linking GFP genetically to any protein of interest, hence making it well observable by fluorescence microscopy [9]. Later on, the sequence of GFP was modified by different mutations, which shifted the color of fluorescence emission to other wavelengths, thereby producing fluorescent proteins that span the entire visible spectrum from blue to red and even beyond [10]. In this respect, the work of Tsien’s lab has to be emphasized as they produced many of the fluorescent proteins that are used nowadays, as well as many fluorescent protein based biosensors. Shimomura, Chalfie and Tsien were awarded the Nobel Prize in Chemistry in 2008 for their discovery. A detailed review on the scientific advantages that came with the discovery of GFP can be found in [11]. A major consequence of the development of spectrally different fluorescent proteins is the fact that appropriate pairs of them can be used as FRET-donors and acceptors, respectively. Furthermore, these spectrally different fluorescent proteins can be linked genetically by a variety of spacers leading to intramolecular FRET. If a spacer in some way responds to a change in the environment, leading to an alteration in the distance between the fluorophores or their fluorescence properties, this will result in a change of the FRET signal. Such a genetically engineered construct can thus serve as FRET-biosensor. Since a great variety of spacers and fluorescent protein pairs can be designed, an excessive diversity of biological processes can be made not only qualitatively visible, but also quantitatively measureable. Certainly, this general concept of FRET-biosensors can be achieved not only with fluorescent proteins, but also with other fluorophores. Nonetheless, our review focusses on genetically encoded fluorescence-protein based FRET-biosensors as this group showed the most vivid development in the last few years.

In this review, we aim to give an overview of currently used fluorescent protein based FRET-biosensors, as well as examples of biological questions that have been addressed with the help of these biosensors. Furthermore, we want to elucidate the basics of designing such a FRET biosensor and we will describe different approaches for their detection and quantification.

## 2. Considerations and Limitations of FRET Based Biosensors

Due to the above-mentioned requirements of fluorescent-protein-based FRET pairs, there is only a specific assortment of appropriate donors and acceptors, which can actually act as a pair. A representative list of fluorescent protein FRET pairs can be found in [12], some of which are occurring in this review and are shown in Table 1.

The first commonly used pair consisted of the two GFP derivatives CFP (cyan) as the donor and YFP (yellow) as the acceptor. Although they are still commonly used, several drawbacks of these two proteins were discovered over the years. YFP shows a very high dependency on its environmental conditions, especially the pH [13], the chloride concentration [14], and the amount of O_2_ that is available during chromophore formation [15]. Although some applications use exactly these drawbacks to design biosensors for these variables (as shown later in this review), it can often lead to a false positive result when a shift in FRET efficiency is not actually produced by the reaction that should be monitored, but rather by a change in environmental factors during the reaction. There are several advanced versions of YFP like Citrine, Venus and Ypet, which show an improved resistance against pH-changes and chloride fluctuations, a better and more robust maturation of the protein, and a higher brightness [16].

**Table 1:** The physical properties of several commonly used fluorescent protein FRET-pairs. An excerpt from Müller *et al.* [12].

Donor	Acceptor	Förster Radius *R_0_* (nm)	Dynamic Range (nm)	Original Source
EBFP	EGFP	4.1	2.1–6.2	[17]
ECFP	EYFP	4.9	2.5–7.3	[17]
mCerulean	Venus	5.4	2.7–8.1	[18,19]
mCerulean	mCitrine	5.4	2.7–8.1	[18]
mTurquoise	mVenus	5.7	2.9–8.6	[19]
EGFP	EYFP	5.6	2.8–8.4	[17]
EGFP	DsRed	4.7(5.8)	2.4–7.1(2.9–8.7)	[20]
EGFP	mRFP1	4.7	2.4–7.1	[21]
Clover	mRuby2	6.3	3.2–9.5	[22]
Dronpa	mCherry	5.6	2.8–8.4	[12]
EYFP	DsRed	4.9	2.5–7.4	[17]
EYFP	mCherry	5.7	2.9–8.6	[23]

CFP on the other hand is much more resistant to environmental conditions. However, it suffers from certain other drawbacks, which render it unsuited for a frequently used FRET measurement technique: monitoring the increase in donor fluorescence upon bleaching of the acceptor. Since the acceptor withdraws energy from the donor, the latter usually gets dimmer in presence of the acceptor. A destruction of the acceptor fluorophore by photobleaching at the specific acceptor excitation wavelength therefore leads to an increase in donor fluorescence, which can be used for a direct calculation of the FRET efficiency provided the acceptor is bleached completely, without any co-bleaching of the donor. Interestingly, more recent experiments have shown that the fluorescence of CFP, and also that of the more novel variant Cerulean, increases in the absence of a FRET acceptor when illuminated at the YFP excitation wavelength that is normally used for acceptor bleaching [24]. This photo-activation effect can lead to false positive FRET signals or an overestimation of the extent of FRET, as the rise in donor intensity is not purely accounted for by the missing of acceptor, but also by the increase due to photo-activation during bleaching. Another factor that has to be considered is the low brightness of the original CFP, having for example a four-fold lower brightness than its counterpart YFP [10]. With a high-sensitivity equipment, this usually is not a problem, or can even be advantageous when used in combination with a highly fluorescent acceptor, leading to a high ratio change with only a little decrease in donor signal. However, it can be a problem when using equipment with a low sensitivity, or very short illumination times, e.g., during timed live-cell experiments. This issue has been addressed by mutations, resulting in many enhanced versions like eCFP, Cerulean [25] and mTurquoise [26].

More recent approaches often use green donors like GFP, eGFP (enhanced GFP) or Clover [22] in combination with a red acceptor like monomeric DsRed [27], RFP [28] or mRuby [29]. These generally outperform CFP and YFP in photostability, brightness, resistance against environmental changes, and dynamic range of distance measurable by FRET [10].

Whichever fluorescent proteins are chosen for a specific application, there are certain factors that should be considered with all of them. First of all, although they are rather small proteins, with a mass of 27 kDa (240AA) for GFP, they are still large enough to be able to impair or alter the function of a protein they are linked to [30]. For most FRET biosensors, this is not an issue as they are external reporters of a pathway that should only respond, and not have any function themselves, but there are some applications where FRET biosensors are bound to cellular structures leading to observations that differ from the actual effect in the unmodified system. Furthermore, it is possible that the sensory domain of the FRET-biosensor affects the biological agent that it should sense for instance by binding to it so that the presence of the biosensor itself is changing the system. Additionally, fluorophores show a tendency to self-association. This can be a major problem, especially with the older variants. More modern mutants tend to be modified in a way to prevent this, therefore often termed monomeric [10].

Another aspect that has to be considered is the problem of photobleaching. This is rarely a problem in short term experiments, but can accumulate to a large bias in long-term studies that observe effects over a longer period of time, especially due to the fact that different fluorophores show widely varying bleaching kinetics [24]. Therefore, in order to obtain reliable results, it is important to determine the bleaching kinetics of the applied FRET pair using proper controls and accounting for it during evaluation.

### 2.1. FRET-Biosensor Design and Considerations

Although the design and improvement of FRET-based biosensors still rely partially on trial-and-error, some main guidelines can be distinguished. In brief, FRET-based biosensors consist of three main parts: the fluorophore pair, the linker and the recognition domain. Importantly, the specificity will be mostly given by the recognition domain (e.g., a phosphorylation substrate sequence). In order to test the specificity of a given probe for the studied molecule, similar molecules can be added in different concentrations [31,32]. If the probe is specific, just the selected molecule will have a significant influence on the FRET efficiency. Since most of the pre-existing biological recognition domains that are used in biosensors have high affinities and might be saturated, it should be considered to perform site-specific mutations to reduce potential saturation problems [33,34,35,36]. Yet not just the recognition domain plays an important role in FRET-based biosensors. The linker region can affect the dipole orientation and the distance between fluorophores and has to be optimized in many cases. However, there is no ideal length for a FRET-biosensor linker. On the one hand, a long and flexible linker has been shown to reduce the basal FRET level due to the increment of the distance between fluorophores and a reduction in the orientation-dependent FRET [37]. On the other hand, a short and rigid linker can maintain a favourable orientation of the fluorophores, which is also important for the FRET efficiency. A main technique to increase the FRET efficiency in an orientation-dependent manner is the circular permutation [31,36,38]. This technique relies on changing the amino acid order without changing the sequenciality itself, thus just changing the place where the starting and ending point of the fluorophore are. Such changes cause a 3D-fluorophore rotation while keeping the same 3D structure, making it a convenient methodology for FRET-based biosensors. Van der Krogt *et al.*, observed that for an optimal FRET-biosensor signal the two beta-barrels of the fluorophores have to be in an antiparallel orientation, promoting fluorophore dimerization [39]. Additionally, Komatsu *et al.*, showed that when long and flexible linkers were placed in the FRET probe, circular-permutated fluorophore variants did not show any difference in the FRET efficiency [37], thus corroborating the above mentioned argumentations. The dimerization between fluorophores is an important parameter that can also be beneficial. When both fluorophores dimerize, the distance between them is reduced, thus considerably increasing the FRET efficiency. However, for a high range between maximum and minimum states, a weak dimerization has been shown to be beneficial [40,41,42]. Moreover, strong dimerization decreases the dynamic range and increases the basal FRET [41]. For a convenient fine-tuning of the dimerization tendency, several mutations for different FRET pairs can be used to increase or decrease it [40,41].

### 2.2. FRET vs. Single-FP Biosensors

Besides FRET-based genetically encoded biosensors, incorporating two or more fluorescent protein variants, there are also sensors available that are based on a single FP. These are usually designed in a way that either the intensity, the excitation profile or the emission profile will change in response to a biochemical alteration.

An example where both FRET-biosensors and single FP sensors are available is for sensing Ca^2+^ concentration levels. One of the very first developed genetically encoded FRET-biosensor was produced in the Tsien lab in order to determine Ca^2+^ levels [43]. The mechanism of this biosensor will be explained more precisely in Section 4.2.4. Nakai *et al.*, developed a single-FP based sensor called GCaMP shortly after in 2001 [44] that aims to measure the same constant. Another example would be the sensing of redox state, as developed by Hanson *et al.* [45].

The great advantage of a single-FP based approach is that the ratio of stimulus strength to fluorescence intensity change tends to be greater as compared to FRET-based ones. However, single-FP based sensors often do not allow a ratiometric detection, and thus a more robust quantification [46], which is inherent to a FRET-based sensor.

Moreover, the design of a single-FP biosensor is tricky. Most modern approaches, including GCaMP, use specific variants of FPs that have been circularly permutated in order to bring the C and N terminal end close to the fluorophore in the center of the barrel structure. The otherwise very stable structure of the FPs can therefore much easier be disrupted by conformational changes. The structure of GCaMP has recently been determined by X-ray crystallography, giving more insight into the function of this type of biosensors [47]. A thorough review of single-FP sensors by Bonnot *et al.*, can be found in [48].

## 3. Measurement and Evaluation of FRET

The occurrence of FRET between two fluorophores has an impact on many spectroscopic and physical properties of the fluorophores that can be measured by different methods. The quantification of FRET has been reviewed in detail many times [4], so this review only gives a very short overview over the different possibilities and their advantages and drawbacks.

### 3.1. Fluorescence Intensity Based Methods

**Figure 3:**
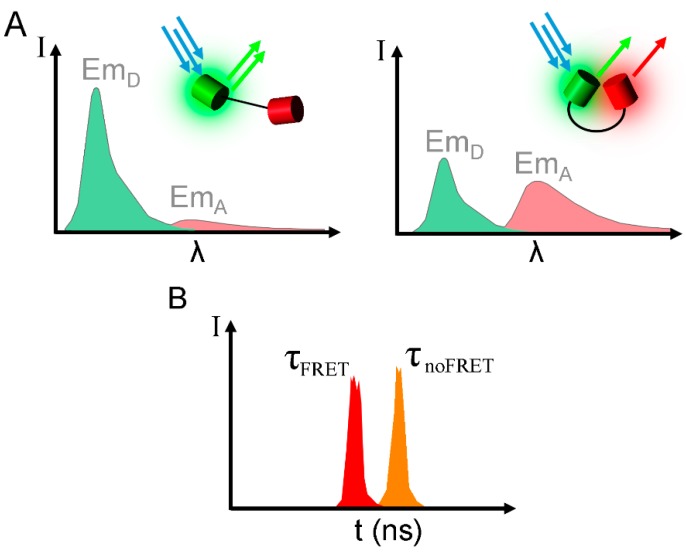
Effects of FRET on the spectroscopic properties. (**A**) FRET produces a change in the emissions intensities of the donor (Em_D_) and the acceptor (Em_A_), clearly measurable as a change in ratio; (**B**) FRET has a characteristic effect on the fluorescent lifetime τ, the delay between excitation and emission.

Techniques based on fluorescence intensities recorded through appropriate filter sets are generally the easiest and most commonly used methods for measuring FRET, as the theory behind it is simple and rather basic equipment is sufficient to determine FRET this way. However, in most cases these methods are not used for an exact quantification of FRET efficiencies but rather for monitoring relative changes. As described earlier, FRET usually leads to the decrease of donor emission and an increase in acceptor emission. This effect can easily be measured with a microscope by using an appropriate setup of spectroscopic filters to differentiate the two excitations and emissions. There are several methods described in the literature to normalize the measured “raw-FRET” (acceptor emission upon donor excitation) to obtain the FRET efficiency [49]. However, as FRET biosensors are mostly uni-molecular, and the concentration of both fluorophores is equal, the more common method to determine alterations in the FRET signal is to measure changes in the ratio of the two emissions (Figure 3A). This is not only the easiest, but also the most versatile method, as it can be applied on a wide variety of equipment, including fluorescence microscopes, plate readers and flow cytometers. It is also suitable for the measurement on live cells, as the measuring method is non-destructive to the fluorophores, except for some bleaching that accompanies longer exposure times, but which can be normalized for with proper controls.

For the measurement of intensity-based methods, there are two different approaches to detect and analyse the resulting fluorescence. The first and classical method is ratiometric imaging, in which glass filters are used to discriminate donor and acceptor fluorescence from each other. While this is a mathematically simple method that is easily standardized, it bears certain drawbacks. First and foremost, not the entire fluorescence signal is detected, as only the signal within the bandwidth of the filters reaches the detector. This potentially reduces signal intensity and therefore sensitivity and signal-to-noise ratio. The alternative approach is spectral unmixing. Here, the entire signal of fluorescence is acquired by the detector, together with the information of the respective wavelength, giving an emission spectrum rather than only the intensity. By comparing this spectrum to the spectra of the lone donor and acceptor, it is possible to calculate the donor and acceptor emission spectra individually. This method therefore overcomes the aforementioned problems, but also brings other advantages. The choice of fluorophores is always limited by the filter setup in ratiometric imaging and FRET-pairs, which show a high overlap in donor emission and acceptor excitation can usually not be used due to the high excess of bleed-through. In spectral unmixing, even very closely related spectra can effectively be discriminated, opening the field up for a whole range of commonly not used, but highly effective FRET-pairs [50].

Another interesting aspect of spectral unmixing is the applicability to more than one biosensor at the same time. There are several examples where two biosensors have been used at the same time. Su *et al.*, used a combination of a BFP/GFP and a Venus/Orange FRET-biosensor to observe Src and Ca^2+^ signalling simultaneously in living cells [51]. Woehler *et al.*, even demonstrated the use of three different biosensors consisting only of a set of three different fluorophores, in this case CFP, YFP and RFP [52]. They also postulated that an up-scaling of this method to four or even five fluorophores is possible, increasing the number of biosensors and making it possible to assess numerous biological parameters in parallel. Although not genetically encoded, Geissler *et al.*, showed the use of six different dyes in order to determine 5 individual binding events at the same time [53]. This could become very useful for monitoring several biological processes and parameters simultaneously in living cells. Other examples of Biosensors that utilize more than two fluorophores at the same time to measure different factors are Wu *et al.*, in 2006 [54] and Kominami *et al.*, in 2012 [55] who used them to measure the activity of different caspases at the same time, as explained in more detail later in this review.

Two alternative techniques are acceptor- and donor-photobleaching. Although common in other FRET applications, these are very rarely seen in FRET biosensors, as the measurement is not repeatable at different time-points in the same sample due to the photo-destruction of one of the two fluorophores by bleaching during the measurement. Additionally, these methods are prone to photoactivation and photoswitching effects of certain fluorophores, as described earlier in Section 2 [24].

### 3.2. Fluorescence Lifetime Based Methods

When a fluorophore is illuminated, it remains in an excited state for about 10^−9^ to 10^−8^ s (1–10 ns), before emitting the energy as measurable photons. This delay is called fluorescence lifetime. The exact length of this depends on the kinetics of all radiative and non-radiative processes that bring the excited molecule back into the ground state, and is characteristic for each fluorophore, like a spectroscopic fingerprint. The addition of FRET as an alternative route of relaxation increases the speed of this process, effectively shortening the fluorescence lifetime. Using a detector with a very high time-resolution makes it possible to determine this difference of lifetime in the presence and absence of FRET exactly, allowing the calculation of FRET efficiency from this difference (Figure 3B) according to Equation (4), where τ_*DA*_ and τ_*D*_ are the lifetimes of donor fluorescence in the presence or absence of acceptor:

(4) $$E_{FRET}=\frac{\tau_{DA}}{\tau_{D}}$$

Fluorescence lifetime imaging microscopy (FLIM) is very popular in FRET studies as it overcomes many of the problems that are associated with intensity-based approaches like the dependence on donor saturation and the importance of a unified setup throughout all measurements. It is also independent of local concentration, the local excitation intensity, or the local fluorescence excitation light intensity [56]. It is also suitable for measurement of live cells with a good time-resolution [57]. However, besides these physical factors of measurement, it is prone to changes in chemical parameters, like pH, polarity, temperature or the refractive index of the medium [58]. But with these factors accounted for, correctly done FLIM is considered the most accurate way to determine FRET, as it goes aside emission intensity measurements but allows examination of the physical micro-environment of the protein with a very high accuracy. The question that remains is the practicability of this technique for FRET-biosensors, as the here-mentioned biosensors are unimolecular, always expressing equal concentrations of all fluorophores. Therefore, the biggest advantages of FLIM, namely the independence of concentration- and intensity variations, is negated, leaving the question whether the remaining advantages justify the complexity of equipment and measurement technique.

### 3.3. Instrumentation for FRET-Measurements

There is a wide variety of instrumentation available for the measurement of FRET, which is applicable to different sets of experiments. First, a user has to determine the aim of his or her experiment. If it is important to determine the localisation of the Biosensor signal, a microscope with a good resolution is necessary. If the entire cell, or even only an average of many cells is of interest, than equipment for Flow-cytometry or a plate reader might be more suitable due to a usually much faster measurement.

In microscopy, a very important factor is the diffraction limit d. This value determines the distance at which two signals can be spatially resolved from each other, *i.e.*, the distance at which two dots can be discriminated as two dots rather than one:

(5) $$d=\frac{\lambda }{2\times N.A.}$$

(6) N.A.=n×sinα

The diffraction limit is dependent on the wavelength of the measured electromagnetic radiation λ, and the numerical aperture *N.A.* of the used objective. The *N.A.* was first defined by Ernst Abbe [59] and is a common quality criteria of objectives. It is determined by the refraction index *n* of the medium between the objective and the sample, and half the objective opening angle α.

These formulas directly apply to the basic form of microscopy, wide-field microscopy. This is the very classical type of microscope, where the entire sample is illuminated by a source of radiation, and the fluorescence that comes off the sample is collected on a single photo-detector chip. While this method has the advantage of usually being very fast and easy to execute with relatively low-cost equipment, its resolution and signal-to-noise ratio is surpassed by more advanced techniques.

One of these advanced techniques is confocal laser scanning microscopy (CLSM). Here, a laser is used to excite one point of the sample after the other, collecting the resulting fluorescence with a high-sensitivity detector, usually a photomultiplier tube (PMT). This guarantees not only a much higher resolution, but also an increased sensitivity and signal-to-noise ratio compared to conventional microscopy. Another factor is the confocal measurement, which introduces depth-selectivity, removing signal from outside of the focal plane by simply using a pinhole aperture in front of the detector, again increasing “sharpness” of the resulting picture as well as signal-to-noise ratio. However, laser-scanning microscopes tend to be very slow as their speed is limited by the galvanometer mirrors that determine the position of the laser, and a single picture takes a much longer time compared to the classical method. Newer advances in these microscopes have tried to re-engineer these systems in order to obtain a much faster measurement of up to 30 frames per second. However, this again goes on the cost of sensitivity, and the end-user always has to make a compromise between speed and image-quality.

A variation to laser scanning microscopes are multiphoton fluorescence microscopes. These apply a specific illumination of a longer wavelength in order to excite an electron within the sample with two photons at the same time, each containing half the energy needed, resulting in an excitation, and hence an emission of a wavelength that is lower than the excitation wavelength, *i.e.*, producing an anti-stokes shift. The aperture pinhole is not needed in this technique, as depth-selectivity is introduced due to the focus point of the excitation wavelength. Due to the much softer, longer wavelength of excitation, this method is usually very gentle on the sample, leading to a highly reduced bleaching outside of the focal plane. The excitation can also pierce much deeper into biological samples due to the higher penetration of long-wavelength radiation.

As mentioned earlier, an alternative would be the use of a fluorescent measurement system that does not produce a resolved picture, but measures the fluorescent state in a bigger unit, e.g., single objects in flow cytometry, or the entire sample in fluorometry.

**Figure 4:**
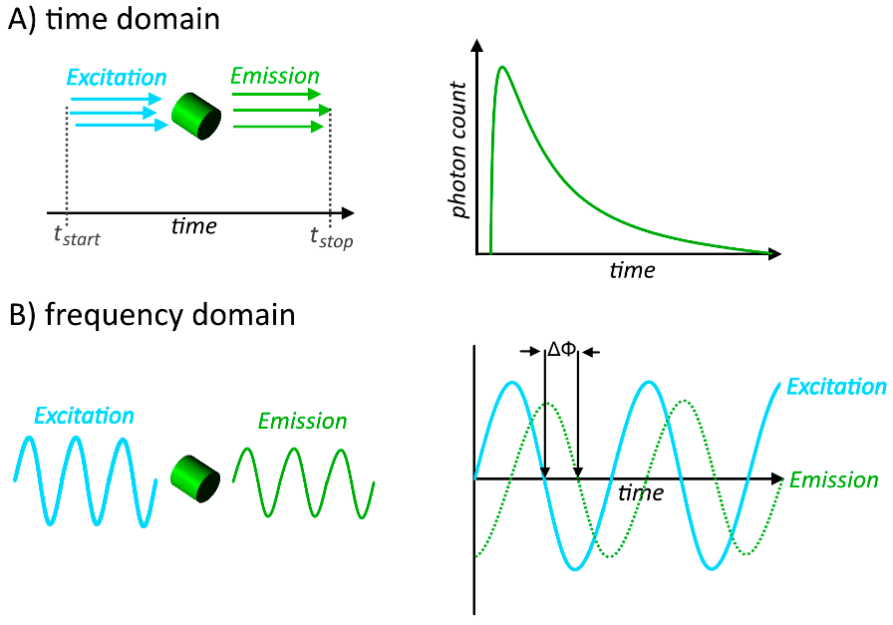
The basic measuring principle of fluorescence lifiteme in (**A**) time-domain, where the time between the exciting light pulse, and emission detection is measured in multiple single measurements to obtain a specific photon count curve; and (**B**) frequency domain, in which the delay in the oscillation of the electromagnetic wave is determined.

All of these methods can be applied to both, intensity based measurements or FLIM measurements. However, FLIM requires a very specific equipment in order to sense the very short lifetime, making it a non-trivial methodology. There are two common ways in which fluorescent lifetime is obtained in FLIM measurements, the first being measurement in the time domain, also called time-correlated single photon counting (TCSPC) [60], in which the time between the excitation laser pulse, and the arrival of the emission at the detector is measured. This is then repeated for several measurements, obtaining a frequency diagram of photon counts, which is specific for the present state of the fluorophore. The second way is to apply a modulated laser for excitation and measurement in the frequency domain [61], in which the delay in the oscillation of the electromagnetic wave is determined. The basic principle of these two measurement methods are shown in Figure 4, and reviewed in [62].

In recent years, especially wide-field-FLIM has gained some attention, as is can be applied by using very inexpensive light-emitting-diodes (LED) as a light source for either time-domain mode, using pulsed excitation, or frequency-domain mode with phase-sensitive detection [63]. While this results in a method with a very high acquisition speed, it lacks the accuracy of measurements in a laser-scanning FLIM system, as the emission is only sampled very briefly, leading to a low photon count on the emission side. Nevertheless, this could make the method of FLIM much more user-friendly, with a low cost and equipment expense, making it usable for many laboratories, and also justifying the use with FRET-biosensors to a greater extend [58].

## 4. Types of FRET Biosensors and Their Practical Applications

Fluorescent protein FRET pairs have been used for a wide array of applications and it is hard to draw an exact line, when such a method is a biosensor, and when not. In this review, we define FRET-biosensors as single encoded proteins that are able to indicate a biological variable by a shift in FRET signal. These are commonly termed intramolecular FRET probes, as both the donor and acceptor are part of the same protein. In contrast, intermolecular FRET would occur between two fluorescently-tagged proteins, which are in close proximity, and is therefore most commonly used to determine interaction of proteins. In the following section, we also classify FRET biosensors by their way of transforming a biological change into a change in FRET efficiency. A list of all biosensors that are covered in this review can be found in Table 2.

### 4.1. Cleavage Based FRET-Biosensors

**Figure 5:**
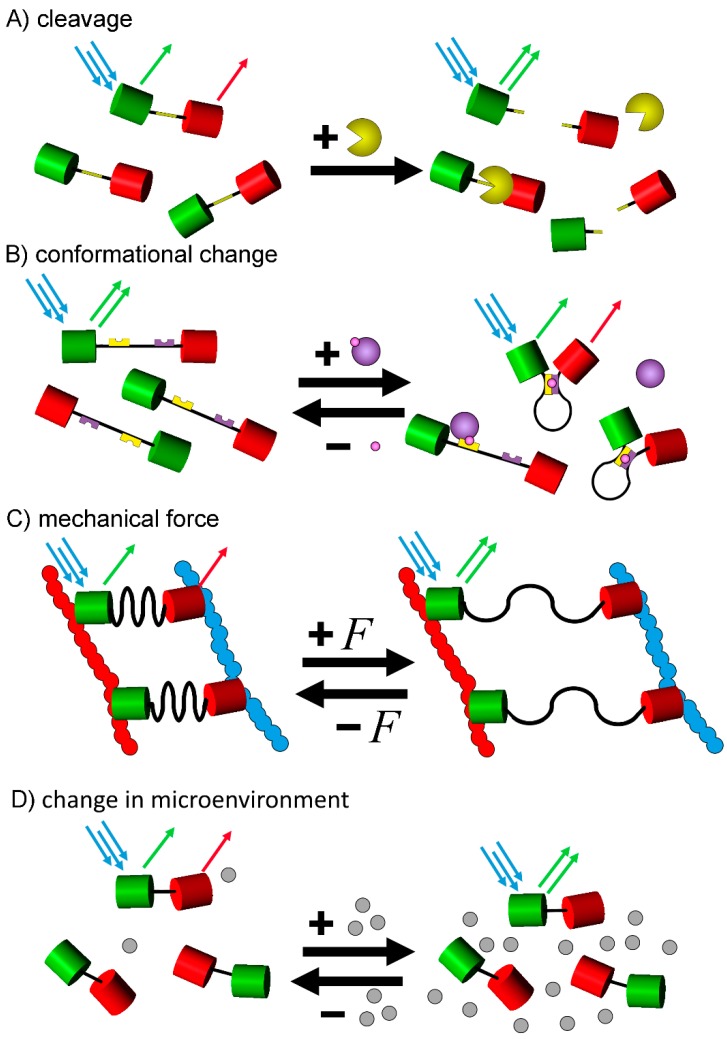
The different types of fluorescent-protein based FRET-biosensors. FRET biosensors indicate a biological change through a change in the FRET signal caused by an alteration of the donor-acceptor distance by (**A**) cleavage of a linker domain; (**B**) a conformational change due to internal alterations or (**C**) a conformational change due to the effect of an external mechanical force. Furthermore, the FRET signal might also be altered (**D**) by a change of the fluorescence properties triggered by a change of the surrounding environment of the fluorophores. The given schemes are only basic examples and many biosensors include alterations to these basic principles, e.g., for conformational change based sensors, the process can be inverted with a high FRET signal at resting state, and the decrease of said signal after posttranslational modification, or the fluorophore pair of a biosensor could be integrated between sensor domains in the middle of a construct.

Possibly the simplest method is based on the cleavage of a specific site within the FRET biosensor. Such a sensor usually consists of a FRET pair that is connected via a short, cleavable linker sequence. In its intact, un-cleaved form, this sensor shows a significant FRET signal due to the short distance between donor and acceptor. Upon the activation of an enzyme that is able to cut a specific sequence that is built into the linker, the spacer is cleaved, the fluorophores separate, and the FRET effect is eliminated, as the two cleavage products can diffuse independent from each other, leading to a shift from acceptor emission to donor emission (Figure 5A). 

While these are a very useful type of FRET-biosensors, they have one major drawback compared to other types, which is the irreversibility of the cleavage. Cleavage based biosensors can sense a change only once by losing their ability to produce FRET, whereas other types remain intact and are often reversible, showing a transformation in both directions as well as often a gradual change of the signal in contrast to the binary type of signal generated by cleavage-based biosensors. Therefore, the latter type is mostly used to determine the activation of specific proteases, often following the stimulation of a pathway.

**Table 2:** A list of original FRET-sensors covered in this review.

		Target	Fluorophores	First Author, Year	*Ref.*
**Cleavage**					
	Apoptosis	Caspase-3	BFP, GFP	Xu, 1998	[64]
		Caspase-3	CFP, YFP	Tyas, 2000	[65]
		Caspase-8 & -9	CFP, Venus	Takemoto, 2003	[66]
		Caspase-3 & -6	CFP, YFP, mRFP	Wu, 2006	[54]
		Caspase-3 & -8	CFP, YFP	Bozza, 2014	[67]
		Caspase-3 & -8	seCFP, Venus, mRFP1	Kominami, 2012	[55]
		Bid	CFP, YFP	Onuki, 2002	[68]
	Necroptosis	RIPK1 & RIPK3	-	Sipieter, 2014	[69]
	Autophagy	Atg4A & Atg4B	CFP, YFP	Li, 2012	[70]
	ECM-remodelling	MT-MMP1	Ypet, ECFP	Ouyang, 2008	[71]
		MT-MMP1	Ypet, ECFP	Lu, 2011	[72]
		MT-MMP1	Orange2, Cherry	Eichorst, 2012	[73]
**Conformational Change**					
	Cell division	CyclinB1-Cdk1	mCerulean, Ypet	Gavet, 2010	[74]
	Signal transduction	AKT	ECFP, Ypet	Miura, 2014	[75]
		AKT-PDK1	CFP, YFP	Yoshizaki, 2007	[76]
		ERK	ECFP, Ypet	Kamioka, 2012	[77]
	Mechano Transduction	FAK	ECFP, Ypet	Seong, 2013	[78]
		Src	ECFP, EYFP	Wang, 2005	[79]
	Metabolite Quantification	ATP	mseCFP, cp173-mVenus	Imamura, 2009	[31]
		ATP	GFP, OFP	Vevea, 2013	[80]
		Glucose	EYFP, ECFP	Fehr, 2003	[32]
		Lactate	mTFP, Venus	San Martin, 2013	[81]
		Ca^2+^	BFP, GFP or CFP, YFP	Miyawaki, 1997	[43]
	Drug Efficacy	BCR-ABL	M1Venus, ECFP	Mizutani, 2010	[82]
		Src	ECFP, EYFP	Nobis, 2013	[83]
	T-cell interaction	ZAP-70	CFP, YFP	Randriamampita, 2008	[84]
		Lck	ECFP, EYFP	Paster, 2009	[85]
**Mechanical Force**					
	Focal Adhesion	Vinculin	mTFP1, Venus	Grashoff, 2010	[86]
	Fluid Shear Stress	VE-cadherin PECAM-1	mTFP1, Venus	Conway, 2013	[87]
		E-cadherin	mTFP1, Venus	Borghi, 2012	[88]
**Changes in the Micro-Environment**					
	Oxygen & ROS	Oxygen	YFP, FbFP	Potzkei, 2012	[15]
		ROS	ECFP, EYFP	Bernardini, 2015	[89]
	pH	pH	GFP, YFP	Awaji, 2001	[90]
		pH	ECFP, EYFP	Urra, 2008	[13]

#### 4.1.1. Apoptosis

One of the most prominent applications of cleavage-based biosensors is for the specific detection of apoptosis. Cell apoptosis is one of the two major forms of cell death, the other being the much less organized necrosis [91]. Apoptosis, as the programmed suicide of cells, plays a very important role in almost all developmental processes, the maintenance of homeostasis, and the reaction to diseases and stress [91]. A central group of proteins during apoptosis is a family of cysteine proteases termed caspases [92,93]. Upon the activation of apoptosis in cells via a death inducing signaling complex, regulatory caspases like caspase-8 are activated. Caspase 8 cleaves the inactive pro-caspase-3 and renders it active [94,95]. Caspase-3 is targeted against the specific sequence Asp-Glu-Val-Asp (DEVD) [96]. The cleavage of the DEVD sequence is therefore a very specific indicator for apoptosis, as necrosis does not activate caspase-3.

The first biosensor that utilized this principle was developed by Xu *et al.*, in 1998 [64]. They simply linked BFP (blue) and GFP with a linker, which contained the DEVD sequence. Upon induction of apoptosis, the protein was cut and the FRET signal disappeared. Tyas *et al.*, used this principle in 2000 to determine the kinetics of caspase 3 activation during apoptosis [65]. They transfected cells with a CFP-DEVD-YFP construct and treated the cells with the apoptosis inducing agent staurosporine. They found that, while the onset of activation had a long delay of ~2 h, the activation of caspase-3 after this delay was completed in ~5 min. Another group came to very similar results, using tumour necrosis factor α (TNFα), staurosporine and etoposide [97].

Caspase-3, which is seen as “effector caspase” at the end of a cascade of proteolytic enzymes, is not the only protein of the caspase family that has been analysed in this way. Caspase 8 and 9, which are considered so-called “initiator-caspases” are known to cut the LEHD sequence, which was the basis for the construction of a CFP-LEHD-Venus construct to measure specifically the initiator enzymes of this proteolytic reaction cascade [66]. In general, it is also possible to determine more than one cleavage with a single FRET biosensor. This has been shown by the construction of a construct containing DEVD and VEID (cleavage site for caspase-6) domains, linked to CFP, YFP and mRFP [54], and one with DEVD and IETD (cleavage site for caspase-8) domains, linked to CFP and YFP [67], or CFP, Venus and mRFP1 respectively.

These reporters are very useful, as apoptosis occurs in many important biological processes. One specific application is verification of the efficacy and high throughput screening of anti-cancer drugs [98]. It has also been used recently to determine the penetration of anti-cancer drugs into a realistic 3D model of a tumour over a long period of time, producing valuable information for the further development of cancer therapies [99]. A very promising and significant application of FRET biosensors is their use in whole-organisms, as shown by Yamaguchi *et al.*, who created a stable transgenic mouse line expressing a FRET biosensor [100]. In general, genomic biosensors are difficult to generate due to the effect that surrounding genes have on their expression, making it either not enough for detection or very variable. This problem could be solved by Yamaguchi *et al.*, by placing two chicken HS4 insulators at each side of a caspase-cleavable FRET construct together with a strong chicken actin (CAG) promoter, which was placed directly in front of the biosensor.

Another process that is tightly linked to apoptosis is the formation of amyloids, clusters of misfolded proteins that precipitate inside and outside of cells, and which are tightly linked to diabetes or neurodegenerative diseases like Alzheimer’s disease [101,102]. Paulsson *et al.*, have used the above-mentioned caspase-3 FRET biosensor to determine the kinetics of apoptosis following the amyloid formation that is present in type 2 diabetes [103]. They found a strong correlation between the formation of amyloid, and the onset of apoptosis, showing the heavy impact of protein misfolding in the progression of this disease.

Upon activation of caspase-8, it not only activates caspase-3, but also the enzyme Bid (PH3 interacting-domain death antagonist) by cleaving the inactive form (22 kDa) into two fragments (p7 and p15) [104]. Bid is a pro-apoptotic member of the Bcl-2 family and can bind to Bax at the outer mitochondrial membrane, leading to the depolarization of mitochondria and release of cytochrome C, which also marks a major event in apoptosis [105,106].

To observe this event, and to study the behaviour of Bid, Onuki *et al.*, generated a CFP-Bid-YFP construct, which is representative of caspase-8 activity [68]. Kominami *et al.*, later postulated a mathematical model, using data gathered with these biosensors, stating that less than 1% of the total caspase-8 must be active to initiate the apoptotic program [107].

#### 4.1.2. Necroptosis

A yet not well-known alternative pathway for programmed cell death is necroptosis. Necroptosis was first described in 2005 [108], and is an important mechanism in the reaction of the immune system to viruses, as well as acute pancreatitis, ischemic injury and septic shock, especially if the apoptosis pathway is pharmacologically or genetically inhibited [109]. Necroptosis lacks the activation of caspases and can therefore be detected by a simple lack of response of conventional FRET-based apoptosis biosensors. However, in 2014, Sipieter *et al.*, described a biosensor that is activated specifically in necroptosis but not in apoptosis [69]. It is based on activation of receptor interacting protein kinases RIPK1 and RIPK3 which are normally kept inactive in apoptosis by caspase-8, but which play an important role in forming the necrosome which drives necroptosis [110]. Necroptosis is seen as a potential alternative for fighting cancer, as necroptotic cells release pro-inflammatory molecules into their extracellular space, like the nuclear factor-high mobility group protein Ba (HMGB1) [111]. This could also potentially overcome resistances to current chemotherapeutic agents, as the sensitivity of cancer cells to necroptosis has already been demonstrated [112].

#### 4.1.3. Autophagy

Another biological process that has been observed with cleavage based FRET biosensors is autophagy, a process that is characterized by the digestion of intracellular components via engulfment with membranes, which then fuse with lysosomes. This process is targeted against superfluous components, damaged cell organelles, misfolded proteins and intracellular pathogens, and is important for keeping the homeostasis of cells intact. A malfunction of autophagy can lead to several diseases, including cancer and neurodegenerative diseases [113,114]. The process involves autophagosomes, organelles with a double membrane, which absorb their targets and subsequently fuse with lysosomes to degrade their content [115].

Autophagosome biogenesis requires the repeated lipidation and delipidation of Atg8, which relies on cleavage of Atg8 by Atg4 [116]. Two biosensors that can determine the amount of autophagy inside cells by utilizing the cleavage site of Atg4A and Atg4B have been developed by Li *et al.*, in 2012 [70], and have later been used by Nguyen *et al.*, to screen several thousand compounds for their inhibitory effect on this pathway [117].

#### 4.1.4. ECM-Remodelling

Another relevant process in which cleavage-based FRET biosensors have been used is the remodelling of the extracellular matrix (ECM). Wound healing, tissue homeostasis, vascularization and embryo development, as well as many pathological events such as inflammation, fibrosis or tumour metastasis, are well-known examples of processes tightly connected with, and dependent on, such ECM remodelling [72].

Matrix metalloproteinases (MMP), a family of zinc-dependent proteases, are reported as decisive players in these processes. In humans, up to 23 different proteases (16 secreted and seven anchored into the membrane) have been described. Although most of them are closely related to tumour progression, some, such as MMP8, have been proposed to be anti-tumourigenic [118,119,120]. Additionally, among all the different types, a subclass of membrane-tethered MMPs (termed MT1-MMP) has recently caught scientists’ attention, since it has been linked as a predominant player in signalling transmission to facilitate metastatic invasions [121,122]. For a better spatio-temporal understanding, Ouyang *et al.*, analyzed MT1-MMP activity by using a FRET-based biosensor composed of a CFP-Ypet FRET-pair, containing a substrate peptide derived from the MT1-MMP cleavage site [71]. Moreover, a PDGFR transmembrane domain was inserted to position the sensor into the extracellular surface of the plasma membrane close to the MT1-MMP, providing a clear advantage over other sensors. Interestingly, after EGF stimulation MT1-MMP activity moved towards the edge of migrating cells (where the EGFR was also present) via a process dependent on an intact cytoskeletal network. Similarly, Eichorst *et al.*, showed MT1-MMP activation along the edge of the cell. In this case, the FP were long-wavelength FRET pairs (Orange2 and Cherry) which improved the probe sensitivity [73]. Lu *et al.*, improved the MT1-MMP biosensor sensitivity further by a factor of five by adding, to a previous eCFP-Ypet FRET-based biosensor, a more specific and sensitive MT1-MMP substrate sequence (AHLR) flanked with flexible linkers. This improved biosensor was then used for quantitatively profiling several breast cancer cell lines, showing a clear correlation between MT1-MMP activity and invasiveness [123]. As for clinical and pharmacological applicability, MMP sensors have been indicated as quick and reliable methods for high-throughput screening of new drug inhibitors and as indicators in tumour progression [123,124].

### 4.2. Conformational Change Based

Biosensor based on a conformational change are a broadly used subclass, which together with the cleavage-based type, represent the vast majority of FRET-based biosensors. They utilize the ability of proteins to change their conformation upon a biological process (Figure 5B). This change could be a posttranslational modification like a phosphorylation for example, or the conformational reaction to an alteration of the environment, like a shift in polarity of the surroundings. One important advantage of such probes is their versatility when it comes to sense specific factors, thus making this type of sensors suitable for a wide range of biological processes. Additionally, conformational-change based biosensors, in contrast to cleavage based ones, are reversible, showing a transformation in both directions.

#### 4.2.1. Cell Division

Cell division is a thoroughly investigated phenomenon, which, due to major subcellular changes, is well suited for the development of FRET-based biosensors. Numerous controlled processes take place throughout the entire cell in a tightly regulated manner. Thus, the visualization of the involved processes in a precise spatiotemporal frame would help to a better understanding of such a primordial event. Gavet *et al.*, studied the Cyclin B1-Cdk1 activity during cell cycle, by using a construct which encoded for a phosphorylation substrate for CyclinB1-Cdk1 linked to a phosphor-amino acid-binding domain (PAABD) and an mCerulean-Ypet fluorophore pair [74]. As PAABDs, one of two common domains is usually used, either a Tryptophan W repeated domain (WW) or a Forkhead-Associated 2 domain (FHA2). Yet, Vandame *et al.*, saw a higher affinity in the WW domain compared to the FHA2 [125]. Thus, once Cyclin B1-Cdk1 phosphorylates the sequence, the PAABD binds to it, bringing both fluorophores closer. This FRET biosensor indicated inactivation of cyclin in the G2 phase and high activity right before the nuclear envelope breakdown [74]. Another player in cell cycle progression is PKA, which has been shown to be highly active near to the chromosomal plate during metaphase and anaphase. A FRET biosensor was employed in this case to demonstrate that PKA-inhibition leads to chromosome misalignment [126].

#### 4.2.2. Signal Transduction

FRET-based sensors are well-suited tools for further analysis of many signalling pathways as well, since highly dynamic and subtle spatiotemporal changes are constantly taking place in cellular networks. In order to capture such minor but important fluctuations as precisely as possible, huge efforts have been made to optimize FRET-based biosensors [37,39,125,127,128]. As already mentioned, distance and orientation between fluorophores play a decisive role. For example, Komatsu *et al.*, developed a biosensor-backbone for kinases and GTPases [37]. Focusing on distance-dependent FRET biosensors, they tested several lengths for a selected linker (between the PAABD and the phospho-substrate). The most adequate length for the linker (termed EV) was between 116 and 224 amino acids, conferring a wider dynamic range and sensitivity, with both parameters being extremely important to visualize variations in protein activity. Several other FRET pairs were tested as well: as donors Turquoise, CyPet and ECFP and as acceptors Venus, circularly-permuted variants of Venus, mCitrine and YPet. In this case, the circularly-permuted variants showed no effect as expected, since the long and flexible linker annulled the orientation-dependent FRET. The largest gain in FRET/CFP was observed for the ECFP-YPet and Turquoise-YPet pairs.

Tracking proteins and their activities with subcellular resolution is a main step further to a better understanding of signalling pathways. A common approach to elucidate protein activity dynamics is to target the same biosensor to different subcellular compartments [75,129,130]. Miura *et al.*, modified the backbone of an AKT sensor and targeted it to membrane rafts, non-raft areas, as well as nuclear and mitochondrial membranes [75]. To that end, the biosensor, coupled with Ypet and eCFP fluorophores, included different localization signals such as a nuclear export signal (NES), nuclear localisation signal (NLS), the plasma-membrane localisation signal H/K-Ras CAAX or a mitochondrial localisation signal (mito). The results showed considerably different kinetics in AKT activity, not just between the locations but also between different cell types upon EGF stimulation. Yoshizaki *et al.*, checked the AKT-PDK1 complexes using a YFP-CFP FRET pair, which followed the same spatio-temporal kinetics than the PtdIns(3,4) distribution, which mainly occurs in the nascent lamellipodia [76].

FRET-based biosensors provide great advantages but the development of a sensors can be a time consuming task [37]. This is even more difficult, if biological processes should be studied *in vivo* in intact organisms. Nevertheless, it could be shown that FRET biosensors can also be designed for these highly physiological systems as exemplified by a transgenic mouse line with a genetically encoded FRET-based biosensor for extracellular regulated kinase, ERK (termed EKAREV) [37,77]. The construct, driven by a strong promoter, consists of a Ypet/eCFP pair, a PAABD, the EV linker, a substrate sequence for ERK and a NLS/NES signal. This EKAREV biosensor helped scientists to describe an interesting cell behaviour based on stochastic ERK activity pulses, which propagated from adjacent cells. Furthermore, the frequency of such pulses but not the amplitude correlated with cell proliferation rates [131]. Continuing in that direction, Hiratsuka *et al.*, used the previously mentioned mouse transgenic line to further study such behaviour [132]. More interestingly, they not just linked such ERK activity pulses to skin cell growth, but additionally the frequency of pulses propagated between cells increased when cell division chemicals were applied and decreased when ERK upstream signalling proteins were blocked. Moreover, ERK activity pulses were also found moving out as waves from the edges of skin wounds. This is a typical example of how more precise and cutting-edge technologies can enable scientists to observe biological behaviours that would have never been perceived with traditional techniques.

#### 4.2.3. Mechano-Transduction

Mechano-transduction forces are also known to be highly dynamic events and therefore an interesting field for FRET biosensors. Seong *et al.*, used a phosphorylation-triggered biosensor with a Ypet-eCFP FRET pair for tracking Focal Adhesion Kinases (FAK) activity in different extracellular matrix conditions of stiffness and composition (collagen I and fibronectin) [78]. It was shown that cells cultured on high-stiffness surfaces (e.g., type I collagen or fibronectin) produced higher traction forces (measured by bead displacement). Fibronectin-coated surfaces triggered a tension-dependent fibronectin-mediated FAK activation response through integrin α5 subunit. However, integrin α2 subunit, which mediates the type I collagen FAK activation, was surprisingly tension-independent. In the same field, the regulation of integrin-cytoskeleton interactions through Src was analysed in more detail [79,133]. Wang *et al.*, observed directional propagating waves along the membrane of Src activity after bead mechanical stimulation, by using a eCFP-eYFP FRET pair with a Src substrate [79]. These waves propagated away from the stimulus with a velocity of 18 nm·s^−1^ and interestingly they were suppressed when cytoskeletal inhibitors were added.

#### 4.2.4. Metabolite Quantification

Another relevant application of conformational based biosensors is the quantification of cell metabolites. Due to their fluctuations triggered by an extensive type of factors, FRET biosensors can help to improve their characterization. A major problem of this type of biosensors in general is their limited capability to sense the whole metabolite concentration range. To solve this, either a unique probe with a maximised range of detection can be used, or a collection of probes, which covers the whole range. FRET biosensors have been developed for many metabolites such as ATP, glucose, glutamate, calcium *etc.* [31,32,80,81,134]. Commonly, bacterial domains that are known to recognize the selected metabolites are inserted into the probe. For example, the ybeJ domain has been used for glutamate, LldR for lactate and the ε subunit of the F_o_F_1_ ATP-synthase for ATP. Once the metabolites bind specifically to the catalytic domain, there is a conformational change that affects the FRET efficiency. In 2013, San Martín *et al.*, developed a methodology to obtain a highly sensitive parameter for the Warburg effect [81]. This effect links the high rates of lactate production of many tumour cells to the presence of oxygen. By using a lactate FRET biosensor coupled to TFP and Venus, with a range from 1 μm to 10 mM, they could precisely determine the levels of lactate in glioma cells and astrocytes with normal or inhibited mitochondria, and use that as indicator for the Warburg effect.

Actually, some of the very first genetically encoded FRET biosensors fall into this category. The lab of Tsien for example produced a Ca^2+^ biosensor in 1997, be fusing two fluorophores (BFP/GFP or CFP/YFP) with a calmodulin, and an M13 domain as linker [43]. Binding of Ca^2+^ makes calmodulin wrap around the M13-domain, hence increasing the effect of FRET. These were later improved several times [135,136].

#### 4.2.5. Drug Efficacy

Conformational change-based biosensors were not only used to observe and determine many natural processes like cell cycle progression and division, but also to analyse a variety of immunological events. As mentioned before, a particularly interesting field for FRET-biosensors is the assessment of drug efficacy in cancer patients. Chronic myeloid leukemia (CML) is a very well-studied form of leukemia, and is in almost all cases accompanied by a reciprocal translocation between chromosomes 9 and 22. This produces the so called philadelphia chromosome, generating the protein fusions BCR-ABL and ABL-BCR, which play a major role in pathogenesis of CML [137]. Mizutani *et al.*, generated a FRET biosensor which linked m1Venus and eCFP with a CrkL linker [82], which is the most characteristic substrate for BCR-ABL [138]. Upon the phosphorylation of CrkL, the conformation changes, increasing the FRET efficiency by 80%. With this biosensor, they could easily assess the efficacy of common protein kinase-inhibiting anti-cancer drugs like imatinib (IM), or second-generation alternatives like nilotinib (NL) and dasatinib (DS). Another type of cancer is pancreatic ductal adenocarcinoma (PDAC) which counts as one of the most lethal forms of human cancer, with 90% of patient deaths occurring within one year after diagnosis [139]. Elevated expression and activity of Src has been demonstrated in many different types of invasive tumours, and leads to an escape from apoptosis and an enhancement of proliferation [140]. A connection between Src upregulation and survival rate has been shown in human PDAC [141]. Src is known to regulate the interaction between integrin and the cytoskeleton [142], which is essential for the mechanical transduction of signals [143]. This has been used as rationale by Wang *et al.*, who produced a FRET-biosensor that allowed the imaging and quantification of Src-activation in living cells [79]. They used it to determine the reaction of Src on the application of a physical force to human umbilical vein endothelial cells (HUVEC), making it possible to observe mechano-transduction in a spatiotemporal manner. Nobis *et al.*, adapted this biosensor to assess drug delivery and efficacy in live PDAC [83]. They could observe a clear switch in Src activity on the invasive borders of the tumour upon the treatment with dasatinib, dependent on the proximity of cells to the host vasculature. They showed that FRET-biosensors can be used to precisely map regions of poor drug-targeting efficiency within tumour microenvironments.

#### 4.2.6. T-Cell Interaction

Another thoroughly investigated part of the human immune system is the interaction of the T-cell receptor with antigen presenting cells (APCs). The presentation of an antigen by APCs leads to the activation of a signalling cascade involving the Syk family tyrosine kinase ZAP-70, which further leads to the phosphorylation of multiple tyrosine residues in the adaptor protein Linker for Activated T cells (LAT). This serves as a docking site for many other proteins, resulting in the assembly of a signalling complex that is crucial for TCR-mediated signal transduction [144]. ZAP-70 is also proposed to be involved in feedback loops, which aim at setting a threshold to TCR activity [145].

To investigate this crucial protein, Randriamampita *et al.*, generated the FRET biosensor ROZA (reporter of Zap-70 activity), which consists of sequences derived from mouse LAT and its respective Src homology (SH) 2 domain, linked to CFP and YFP as a FRET-pair [84]. Upon phosphorylation of LAT by Zap-70, it binds to the SH2 domain, forcing it into a conformation that increases the FRET signal. They could determine the activity of ZAP-70 during TCR-mediated signalling and interestingly found the activation not only at the TCR-APC interface pole (synapse), but also at the opposite side (anti-synapse). This biosensor was recently further developed and improved by Cadra *et al.* [146].

Another part of the TCR-APC signalling pathway is Lck (part of the Src family of tyrosine kinases), involved in the very early wave of tyrosine phosphorylation of immunoreceptor tyrosine-based activation motif (ITAMs), which are intracellular parts of the CD3 and ζ-chains of the TCR-complex, that allows binding of ZAP-70. Paster *et al.*, used the full Lck backbone to create a FRET biosensor to observe different conformations of Lck during signalling [85]. Interestingly, they found no change in conformation, although the biosensor was proven to be sensitive to specific changes.

### 4.3. Mechanical Force Based

The three-dimensional structure of a protein can be changed not only by modifying the protein itself, but also by applying a mechanical force from the outside. A good example for this would be the proteins contained in spider silk. These often feature helical segments which can stretch out to a great extent, giving the thread its elasticity [147]. Mechanical forces like tension are not only a stress to cells, but play a central role in many developmental, physiological and pathological processes, especially regarding the transduction of signals [148].

#### 4.3.1. Focal Adhesion

Focal adhesions (FAs) are physiological structures that anchor cells by connecting the actin cytoskeleton to the extracellular matrix (ECM). Mechanical tension plays a particularly important role in these structures [149]. FAs are directly responding to tension by enlarging, shrinking or even disassembling, depending on the applied force [150].

For a long time, it was almost impossible to measure the exact forces that act on these sites until FRET sensors were developed which can measure them with pico-Newton sensitivity [86,151]. These sensors were built by combining a short linker sequence, derived from the aforementioned spider silk proteins, which can act as a nano-sized spring like structure, with a FRET pair at the two ends. While a relaxed spring would produce a strong FRET signal, applying tension to the sensor increases the distance between donor and acceptor by stretching the spring, therefore decreasing the extent of FRET (Figure 5C). By connecting those sensors with proteins that stand under high force, the force that acts on the protein can be determines precisely. Grashoff *et al.*, applied this method to vinculin [86], which forms an important part of focal adhesion by directly connecting integrins, which reside in the cell membrane and interact with the ECM, with the actin cytoskeleton of the cell [150]. They could show that the force across vinculin in stable FAs is ~2.5 pN. They most interestingly showed that the recruitment of vinculin to sites of increased tension is controlled independently of the transmission of force via vinculin.

#### 4.3.2. Fluid Shear Stress

In order to determine the mechanical forces that are acting on the cells due to the fluid shear stress (FSS) of the natural blood flow, Conway *et al.*, modified the above mentioned sensor design in order to measure the stress across VE-cadherin and PECAM-1 [87], which are parts of a mechanosensory complex, occurring in cell-cell junctions [152,153]. They demonstrated that the tension within cell-cell junctions is quite strong at resting conditions, and decreases within less than 30 s after onset of shear stress. These results argued against a common model of passive force transmission through the junctions, and rather showed that flow triggers cytoskeletal remodelling which alters the forces across junctional receptors. Borghi *et al.*, also utilized this biosensor method to show that E-cadherin is not only important at cell-cell junctions, but that it transduces mechanical forces throughout the entire cell surface [88].

A more detailed review on the measurement of tension across proteins can be found in [154].

### 4.4. Sensors for Micro-Environmental Changes

While the previous three categories of biosensors produce a decrease or increase in FRET by changing the distance between donor and acceptor, the fourth category utilizes the sensitivity of some fluorophores to certain environmental conditions [10] (Figure 5D). As described earlier, the fluorescence of YFP is especially dependent on its surroundings, making it a popular choice for these biosensors.

#### 4.4.1. Oxygen & ROS

Oxygen is one of the basic building blocks of life, and many proteins not only need it in their molecular structure, but also depend on a certain concentration of cellular oxygen during maturation in order to function correctly. So do many fluorescent proteins [16], which has led to the development of hypoxia-sensitive biosensors by combining an oxygen-insensitive fluorophore as the donor with an oxygen sensitive fluorophore as the acceptor. The lower the oxygen level is the less energy is absorbed by the acceptor, leading to a diminishing of FRET signal that is directly relatable to the state of hypoxia in a cell. Potzkei *et al.*, have demonstrated this principle by coupling YFP with a short linker to a Flavin mononucleotide (FMN)-binding fluorescent protein (FbFP) which is independent of oxygen levels [155]. They have used this biosensor in batch processing of Escherichia coli to demonstrate the onset and progression of hypoxia over its full time course. With a different approach, Bernardini *et al.*, recently demonstrated a FRET based biosensor that is sensitive to reactive oxygen species (ROS) by using the redox-dependent regulatory domain of the bacterial heat shock protein HSP33 as a linker [89]. As ROS are involved in many pathological processes, this sensor might become a very useful tool to assess the oxidative state within a cell in many different conditions.

#### 4.4.2. pH

Another biological variable that has been shown to impact fluorescence is the pH level [156]. Awaji *et al.*, have produced and evaluated a pH-sensitive biosensor consisting of GFP and YFP [90]. They demonstrated that they could not only show pH changes in the cytosol and nucleus, but that it was also possible to tether this probe to a membrane protein, making it possible to measure pH changes close to the cellular membranes. Urra *et al.*, generated a similar sensor, but they bound it to the sequence of the platelet-derived growth factor transmembrane domain (PDGFR-TM), which located the sensor on the extracellular surface [13]. With this sensor, they were able to determine the dynamics of pH in extracellular microdomains, a region which is poorly studied and which has often been postulated to be important for the activity of various membrane-bound proteins like receptors, transporters, ion channels and enzymes [13].

## 5. Conclusions/Outlook

FRET provides an incredibly powerful tool, allowing the qualitative and quantitative determination of interactions between molecules or conformational changes. It has been used to assess the dynamics and spatio-temporal characteristics of numerous pathways and interactions in living organisms. However, FRET is often hard to interpret and is victim to certain biases that need proper normalization and controls. FRET biosensors are tools that make use of the FRET effect so as to make biological variables easily and non-invasively measurable by transforming them into a shift in FRET efficiency. The measurement of such variables, especially in living tissues, will become more and more important for a better understanding of the complex signalling cascades and networks that regulate sophisticated biological systems. The advance of the concept towards the measurement of multiple FRET-biosensors at the same time in a single sample will enable scientists to measure a variety of biological processes or parameters simultaneously allowing for multi-factorial analyses at high temporal and spatial resolution. We are convinced that these approaches will lead to a better understanding of signalling networks and their feedback circuits providing a basis for more specific targeting of relevant pathological signalling hubs in the future.

## References

[1] Stokes G.G. (1852). On the change of refrangibility of light. Philos. Trans. R. Soc. Lond..

[2] Förster T. (1948). Zwischenmolekulare energiewanderung und fluoreszenz. Annalen der Physik.

[3] Lovell J.F., Chen J., Jarvi M.T., Cao W.G., Allen A.D., Liu Y., Tidwell T.T., Wilson B.C., Zheng G. (2009). FRET quenching of photosensitizer singlet oxygen generation. J. Phys. Chem. B.

[4] Shrestha D., Jenei A., Nagy P., Vereb G., Szollosi J. (2015). Understanding FRET as a research tool for cellular studies. Int. J. Mol. Sci..

[5] Yang F., Moss L.G., Phillips G.N. (1996). The molecular structure of green fluorescent protein. Nat. Biotechnol..

[6] Shimomura O. (2009). Discovery of green fluorescent protein (GFP) (nobel lecture). Angew. Chem. Int. Ed. Engl..

[7] Prasher D.C., Eckenrode V.K., Ward W.W., Prendergast F.G., Cormier M.J. (1992). Primary structure of the aequorea victoria green-fluorescent protein. Gene.

[8] Chalfie M., Tu Y., Euskirchen G., Ward W.W., Prasher D.C. (1994). Green fluorescent protein as a marker for gene expression. Science.

[9] Tsien R.Y. (1998). The green fluorescent protein. Annu. Rev. Biochem..

[10] Olenych S.G., Claxton N.S., Ottenberg G.K., Davidson M.W. (2007). The fluorescent protein color palette. Curr Protoc Cell Biol..

[11] Schmid J.A., Neumeier H. (2005). Evolutions in science triggered by green fluorescent protein (GFP). Chembiochem.

[12] Muller S.M., Galliardt H., Schneider J., Barisas B.G., Seidel T. (2013). Quantification of forster resonance energy transfer by monitoring sensitized emission in living plant cells. Front. Plant. Sci.

[13] Urra J., Sandoval M., Cornejo I., Barros L.F., Sepulveda F.V., Cid L.P. (2008). A genetically encoded ratiometric sensor to measure extracellular ph in microdomains bounded by basolateral membranes of epithelial cells. Pflugers Arch..

[14] Zhong S., Navaratnam D., Santos-Sacchi J. (2014). A genetically-encoded YFP sensor with enhanced chloride sensitivity, photostability and reduced ph interference demonstrates augmented transmembrane chloride movement by gerbil prestin (slc26a5). PLoS ONE.

[15] Potzkei J., Kunze M., Drepper T., Gensch T., Jaeger K.E., Buchs J. (2012). Real-time determination of intracellular oxygen in bacteria using a genetically encoded FRET-based biosensor. BMC Biol..

[16] Shaner N.C., Steinbach P.A., Tsien R.Y. (2005). A guide to choosing fluorescent proteins. Nat. Methods.

[17] Patterson G.H., Piston D.W., Barisas B.G. (2000). Forster distances between green fluorescent protein pairs. Anal. Biochem..

[18] Rizzo M.A., Springer G., Segawa K., Zipfel W.R., Piston D.W. (2006). Optimization of pairings and detection conditions for measurement of FRET between cyan and yellow fluorescent proteins. Microsc. Microanal..

[19] Markwardt M.L., Kremers G.J., Kraft C.A., Ray K., Cranfill P.J., Wilson K.A., Day R.N., Wachter R.M., Davidson M.W., Rizzo M.A. (2011). An improved cerulean fluorescent protein with enhanced brightness and reduced reversible photoswitching. PLoS ONE.

[20] Erickson M.G., Moon D.L., Yue D.T. (2003). Dsred as a potential FRET partner with CFP and GFP. Biophys. J..

[21] Peter M., Ameer-Beg S.M., Hughes M.K., Keppler M.D., Prag S., Marsh M., Vojnovic B., Ng T. (2005). Multiphoton-flim quantification of the eGFP-MRFP1 FRET pair for localization of membrane receptor-kinase interactions. Biophys. J..

[22] Lam A.J., St-Pierre F., Gong Y., Marshall J.D., Cranfill P.J., Baird M.A., McKeown M.R., Wiedenmann J., Davidson M.W., Schnitzer M.J. (2012). Improving FRET dynamic range with bright green and red fluorescent proteins. Nat. Methods.

[23] Akrap N., Seidel T., Barisas B.G. (2010). Forster distances for fluorescence resonant energy transfer between mcherry and other visible fluorescent proteins. Anal. Biochem..

[24] Malkani N., Schmid J.A. (2011). Some secrets of fluorescent proteins: Distinct bleaching in various mounting fluids and photoactivation of cyan fluorescent proteins at YFP-excitation. PLoS ONE.

[25] Rizzo M.A., Springer G.H., Granada B., Piston D.W. (2004). An improved cyan fluorescent protein variant useful for FRET. Nat. Biotechnol..

[26] Goedhart J., van Weeren L., Hink M.A., Vischer N.O., Jalink K., Gadella T.W. (2010). Bright cyan fluorescent protein variants identified by fluorescence lifetime screening. Nat. Methods.

[27] Matz M.V., Fradkov A.F., Labas Y.A., Savitsky A.P., Zaraisky A.G., Markelov M.L., Lukyanov S.A. (1999). Fluorescent proteins from nonbioluminescent anthozoa species. Nat. Biotechnol..

[28] Merzlyak E.M., Goedhart J., Shcherbo D., Bulina M.E., Shcheglov A.S., Fradkov A.F., Gaintzeva A., Lukyanov K.A., Lukyanov S., Gadella T.W. (2007). Bright monomeric red fluorescent protein with an extended fluorescence lifetime. Nat. Methods.

[29] Kredel S., Oswald F., Nienhaus K., Deuschle K., Rocker C., Wolff M., Heilker R., Nienhaus G.U., Wiedenmann J. (2009). Mruby, a bright monomeric red fluorescent protein for labeling of subcellular structures. PLoS ONE.

[30] Ettinger A., Wittmann T. (2014). Fluorescence live cell imaging. Methods Cell Biol..

[31] Imamura H., Nhat K.P., Togawa H., Saito K., Iino R., Kato-Yamada Y., Nagai T., Noji H. (2009). Visualization of ATP levels inside single living cells with fluorescence resonance energy transfer-based genetically encoded indicators. Proc. Natl. Acad. Sci. USA.

[32] Fehr M., Lalonde S., Lager I., Wolff M.W., Frommer W.B. (2003). *In vivo* imaging of the dynamics of glucose uptake in the cytosol of cos-7 cells by fluorescent nanosensors. J. Biol. Chem..

[33] Okumoto S., Looger L.L., Micheva K.D., Reimer R.J., Smith S.J., Frommer W.B. (2005). Detection of glutamate release from neurons by genetically encoded surface-displayed FRET nanosensors. Proc. Natl. Acad. Sci. USA.

[34] Deuschle K., Chaudhuri B., Okumoto S., Lager I., Lalonde S., Frommer W.B. (2006). Rapid metabolism of glucose detected with FRET glucose nanosensors in epidermal cells and intact roots of arabidopsis rna-silencing mutants. Plant. Cell.

[35] Chen H.L., Bernard C.S., Hubert P., My L., Zhang C.C. (2013). Fluorescence resonance energy transfer based on interaction of PII and PIPX proteins provides a robust and specific biosensor for 2-oxoglutarate, a central metabolite and a signaling molecule. FEBS J..

[36] Hires S.A., Zhu Y., Tsien R.Y. (2008). Optical measurement of synaptic glutamate spillover and reuptake by linker optimized glutamate-sensitive fluorescent reporters. Proc. Natl. Acad. Sci. USA.

[37] Komatsu N., Aoki K., Yamada M., Yukinaga H., Fujita Y., Kamioka Y., Matsuda M. (2011). Development of an optimized backbone of FRET biosensors for kinases and gtpases. Mol. Biol. Cell.

[38] Nagai T., Yamada S., Tominaga T., Ichikawa M., Miyawaki A. (2004). Expanded dynamic range of fluorescent indicators for Ca(2+) by circularly permuted yellow fluorescent proteins. Proc. Natl. Acad. Sci. USA.

[39] Van der Krogt G.N., Ogink J., Ponsioen B., Jalink K. (2008). A comparison of donor-acceptor pairs for genetically encoded FRET sensors: Application to the epac camp sensor as an example. PLoS ONE.

[40] Vinkenborg J.L., Evers T.H., Reulen S.W., Meijer E.W., Merkx M. (2007). Enhanced sensitivity of FRET-based protease sensors by redesign of the GFP dimerization interface. Chembiochem.

[41] Kotera I., Iwasaki T., Imamura H., Noji H., Nagai T. (2010). Reversible dimerization of aequorea victoria fluorescent proteins increases the dynamic range of FRET-based indicators. ACS Chem. Biol..

[42] Nguyen A.W., Daugherty P.S. (2005). Evolutionary optimization of fluorescent proteins for intracellular FRET. Nat. Biotechnol..

[43] Miyawaki A., Llopis J., Heim R., McCaffery J.M., Adams J.A., Ikura M., Tsien R.Y. (1997). Fluorescent indicators for Ca2+ based on green fluorescent proteins and calmodulin. Nature.

[44] Nakai J., Ohkura M., Imoto K. (2001). A high signal-to-noise Ca(2+) probe composed of a single green fluorescent protein. Nat. Biotechnol..

[45] Hanson G.T., Aggeler R., Oglesbee D., Cannon M., Capaldi R.A., Tsien R.Y., Remington S.J. (2004). Investigating mitochondrial redox potential with redox-sensitive green fluorescent protein indicators. J. Biol. Chem..

[46] Frommer W.B., Davidson M.W., Campbell R.E. (2009). Genetically encoded biosensors based on engineered fluorescent proteins. Chem. Soc. Rev..

[47] Akerboom J., Rivera J.D., Guilbe M.M., Malave E.C., Hernandez H.H., Tian L., Hires S.A., Marvin J.S., Looger L.L., Schreiter E.R. (2009). Crystal structures of the gcamp calcium sensor reveal the mechanism of fluorescence signal change and aid rational design. J. Biol. Chem..

[48] Bonnot A., Guiot E., Hepp R., Cavellini L., Tricoire L., Lambolez B. (2014). Single-fluorophore biosensors based on conformation-sensitive GFP variants. FASEB J..

[49] Chen H., Puhl H.L., Koushik S.V., Vogel S.S., Ikeda S.R. (2006). Measurement of FRET efficiency and ratio of donor to acceptor concentration in living cells. Biophys. J..

[50] Zimmermann T., Rietdorf J., Pepperkok R. (2003). Spectral imaging and its applications in live cell microscopy. FEBS Lett..

[51] Su T., Pan S., Luo Q., Zhang Z. (2013). Monitoring of dual bio-molecular events using FRET biosensors based on mtagbfp/sfgfp and mvenus/mkokappa fluorescent protein pairs. Biosens. Bioelectron..

[52] Woehler A. (2013). Simultaneous quantitative live cell imaging of multiple FRET-based biosensors. PLoS ONE.

[53] Geissler D., Stufler S., Lohmannsroben H.G., Hildebrandt N. (2013). Six-color time-resolved forster resonance energy transfer for ultrasensitive multiplexed biosensing. J. Am. Chem. Soc..

[54] Wu X., Simone J., Hewgill D., Siegel R., Lipsky P.E., He L. (2006). Measurement of two caspase activities simultaneously in living cells by a novel dual FRET fluorescent indicator probe. Cytometry A.

[55] Kominami K., Nagai T., Sawasaki T., Tsujimura Y., Yashima K., Sunaga Y., Tsuchimochi M., Nishimura J., Chiba K., Nakabayashi J. (2012). *In vivo* imaging of hierarchical spatiotemporal activation of caspase-8 during apoptosis. PLoS ONE.

[56] Lakowicz J.R., Szmacinski H., Nowaczyk K., Berndt K.W., Johnson M. (1992). Fluorescence lifetime imaging. Anal. Biochem..

[57] Hum J.M., Siegel A.P., Pavalko F.M., Day R.N. (2012). Monitoring biosensor activity in living cells with fluorescence lifetime imaging microscopy. Int. J. Mol. Sci..

[58] Ishikawa-Ankerhold H.C., Ankerhold R., Drummen G.P. (2012). Advanced fluorescence microscopy techniques—FRAP, flip, flap, FRET and flim. Molecules.

[59] Abbe E. (1881). Vii.—on the estimation of aperture in the microscope. J. R. Microsc. Soc..

[60] Rinnenthal J.L., Bornchen C., Radbruch H., Andresen V., Mossakowski A., Siffrin V., Seelemann T., Spiecker H., Moll I., Herz J. (2013). Parallelized TCSPC for dynamic intravital fluorescence lifetime imaging: Quantifying neuronal dysfunction in neuroinflammation. PLoS ONE.

[61] Hinde E., Digman M.A., Welch C., Hahn K.M., Gratton E. (2012). Biosensor forster resonance energy transfer detection by the phasor approach to fluorescence lifetime imaging microscopy. Microsc. Res. Tech..

[62] Becker W. (2012). Fluorescence lifetime imaging—techniques and applications. J. Microsc..

[63] Jan Willem B., Antonie J.W.G.V. (2010). Fluorescence lifetime imaging microscopy in life sciences. Meas. Sci. Technol..

[64] Xu X., Gerard A.L., Huang B.C., Anderson D.C., Payan D.G., Luo Y. (1998). Detection of programmed cell death using fluorescence energy transfer. Nucl. Acids Res..

[65] Tyas L., Brophy V.A., Pope A., Rivett A.J., Tavare J.M. (2000). Rapid caspase-3 activation during apoptosis revealed using fluorescence-resonance energy transfer. EMBO Rep..

[66] Takemoto K., Nagai T., Miyawaki A., Miura M. (2003). Spatio-temporal activation of caspase revealed by indicator that is insensitive to environmental effects. J. Cell Biol..

[67] Bozza W.P., Di X., Takeda K., Rivera Rosado L.A., Pariser S., Zhang B. (2014). The use of a stably expressed FRET biosensor for determining the potency of cancer drugs. PLoS ONE.

[68] Onuki R., Nagasaki A., Kawasaki H., Baba T., Uyeda T.Q., Taira K. (2002). Confirmation by FRET in individual living cells of the absence of significant amyloid beta -mediated caspase 8 activation. Proc. Natl. Acad. Sci. USA.

[69] Sipieter F., Ladik M., Vandenabeele P., Riquet F. (2014). Shining light on cell death processes—A novel biosensor for necroptosis, a newly described cell death program. Biotechnol J..

[70] Li M., Chen X., Ye Q.Z., Vogt A., Yin X.M. (2012). A high-throughput FRET-based assay for determination of ATG4 activity. Autophagy.

[71] Ouyang M., Lu S., Li X.Y., Xu J., Seong J., Giepmans B.N., Shyy J.Y., Weiss S.J., Wang Y. (2008). Visualization of polarized membrane type 1 matrix metalloproteinase activity in live cells by fluorescence resonance energy transfer imaging. J. Biol. Chem..

[72] Lu P., Takai K., Weaver V.M., Werb Z. (2011). Extracellular matrix degradation and remodeling in development and disease. Cold Spring Harb. Perspect. Biol..

[73] Eichorst J.P., Clegg R.M., Wang Y. (2012). Red-shifted fluorescent proteins monitor enzymatic activity in live ht-1080 cells with fluorescence lifetime imaging microscopy (flim). J. Microsc..

[74] Gavet O., Pines J. (2010). Progressive activation of cyclinb1-cdk1 coordinates entry to mitosis. Dev. Cell.

[75] Miura H., Matsuda M., Aoki K. (2014). Development of a FRET biosensor with high specificity for akt. Cell. Struct. Funct..

[76] Yoshizaki H., Mochizuki N., Gotoh Y., Matsuda M. (2007). Akt-pdk1 complex mediates epidermal growth factor-induced membrane protrusion through RAL activation. Mol. Biol Cell.

[77] Kamioka Y., Sumiyama K., Mizuno R., Sakai Y., Hirata E., Kiyokawa E., Matsuda M. (2012). Live imaging of protein kinase activities in transgenic mice expressing FRET biosensors. Cell Struct. Funct..

[78] Seong J., Tajik A., Sun J., Guan J.L., Humphries M.J., Craig S.E., Shekaran A., Garcia A.J., Lu S., Lin M.Z. (2013). Distinct biophysical mechanisms of focal adhesion kinase mechanoactivation by different extracellular matrix proteins. Proc. Natl. Acad. Sci. USA.

[79] Wang Y., Botvinick E.L., Zhao Y., Berns M.W., Usami S., Tsien R.Y., Chien S. (2005). Visualizing the mechanical activation of SRC. Nature.

[80] Vevea J.D., Wolken D.M., Swayne T.C., White A.B., Pon L.A. (2013). Ratiometric biosensors that measure mitochondrial redox state and atp in living yeast cells. J. Vis. Exp..

[81] San Martin A., Ceballo S., Ruminot I., Lerchundi R., Frommer W.B., Barros L.F. (2013). A genetically encoded FRET lactate sensor and its use to detect the warburg effect in single cancer cells. PLoS ONE.

[82] Mizutani T., Kondo T., Darmanin S., Tsuda M., Tanaka S., Tobiume M., Asaka M., Ohba Y. (2010). A novel FRET-based biosensor for the measurement of bcr-abl activity and its response to drugs in living cells. Clin. Cancer Res..

[83] Nobis M., McGhee E.J., Morton J.P., Schwarz J.P., Karim S.A., Quinn J., Edward M., Campbell A.D., McGarry L.C., Evans T.R. (2013). Intravital flim-FRET imaging reveals dasatinib-induced spatial control of src in pancreatic cancer. Cancer Res..

[84] Randriamampita C., Mouchacca P., Malissen B., Marguet D., Trautmann A., Lellouch A.C. (2008). A novel zap-70 dependent FRET based biosensor reveals kinase activity at both the immunological synapse and the antisynapse. PLoS ONE.

[85] Paster W., Paar C., Eckerstorfer P., Jakober A., Drbal K., Schutz G.J., Sonnleitner A., Stockinger H. (2009). Genetically encoded forster resonance energy transfer sensors for the conformation of the src family kinase lck. J. Immunol..

[86] Grashoff C., Hoffman B.D., Brenner M.D., Zhou R., Parsons M., Yang M.T., McLean M.A., Sligar S.G., Chen C.S., Ha T. (2010). Measuring mechanical tension across vinculin reveals regulation of focal adhesion dynamics. Nature.

[87] Conway D.E., Breckenridge M.T., Hinde E., Gratton E., Chen C.S., Schwartz M.A. (2013). Fluid shear stress on endothelial cells modulates mechanical tension across ve-cadherin and pecam-1. Curr. Biol..

[88] Borghi N., Sorokina M., Shcherbakova O.G., Weis W.I., Pruitt B.L., Nelson W.J., Dunn A.R. (2012). E-cadherin is under constitutive actomyosin-generated tension that is increased at cell-cell contacts upon externally applied stretch. Proc. Natl. Acad. Sci. USA.

[89] Bernardini A., Brockmeier U., Metzen E., Berchner-Pfannschmidt U., Harde E., Acker-Palmer A., Papkovsky D., Acker H., Fandrey J. (2015). Type i cell ros kinetics under hypoxia in the intact mouse carotid body *ex vivo*: A FRET-based study. Am. J. Physiol. Cell Physiol..

[90] Awaji T., Hirasawa A., Shirakawa H., Tsujimoto G., Miyazaki S. (2001). Novel green fluorescent protein-based ratiometric indicators for monitoring ph in defined intracellular microdomains. Biochem. Biophys. Res. Commun..

[91] Green D.R. (2011). Means to an End: Apoptosis and Other Cell Death Mechanisms.

[92] Miura M. (2012). Apoptotic and nonapoptotic caspase functions in animal development. Cold Spring Harb. Perspect. Biol..

[93] Thornberry N.A., Lazebnik Y. (1998). Caspases: Enemies within. Science.

[94] Budihardjo I., Oliver H., Lutter M., Luo X., Wang X. (1999). Biochemical pathways of caspase activation during apoptosis. Annu. Rev. Cell Dev. Biol..

[95] Wolf B.B., Green D.R. (1999). Suicidal tendencies: Apoptotic cell death by caspase family proteinases. J. Biol. Chem..

[96] Lazebnik Y.A., Kaufmann S.H., Desnoyers S., Poirier G.G., Earnshaw W.C. (1994). Cleavage of poly(adp-ribose) polymerase by a proteinase with properties like ice. Nature.

[97] Rehm M., Dussmann H., Janicke R.U., Tavare J.M., Kogel D., Prehn J.H. (2002). Single-cell fluorescence resonance energy transfer analysis demonstrates that caspase activation during apoptosis is a rapid process. Role of caspase-3. J. Biol. Chem..

[98] Tian H., Ip L., Luo H., Chang D.C., Luo K.Q. (2007). A high throughput drug screen based on fluorescence resonance energy transfer (FRET) for anticancer activity of compounds from herbal medicine. Br. J. Pharmacol..

[99] Anand P., Fu A., Teoh S.H., Luo K.Q. (2015). Application of a fluorescence resonance energy transfer (FRET)-based biosensor for detection of drug-induced apoptosis in a 3d breast tumor model. Biotechnol. Bioeng..

[100] Yamaguchi Y., Shinotsuka N., Nonomura K., Takemoto K., Kuida K., Yosida H., Miura M. (2011). Live imaging of apoptosis in a novel transgenic mouse highlights its role in neural tube closure. J. Cell. Biol..

[101] Glenner G.G. (1980). Amyloid deposits and amyloidosis. The beta-fibrilloses (first of two parts). N. Eng. J. Med..

[102] Jarrett J.T., Lansbury P.T. (1993). Seeding “one-dimensional crystallization” of amyloid: A pathogenic mechanism in alzheimer’s disease and scrapie?. Cell.

[103] Paulsson J.F., Schultz S.W., Kohler M., Leibiger I., Berggren P.O., Westermark G.T. (2008). Real-time monitoring of apoptosis by caspase-3-like protease induced FRET reduction triggered by amyloid aggregation. Exp. Diabetes. Res..

[104] Li H., Zhu H., Xu C.J., Yuan J. (1998). Cleavage of bid by caspase 8 mediates the mitochondrial damage in the fas pathway of apoptosis. Cell.

[105] Wang K., Yin X.M., Chao D.T., Milliman C.L., Korsmeyer S.J. (1996). Bid: A novel bh3 domain-only death agonist. Genes Dev..

[106] Li P., Nijhawan D., Budihardjo I., Srinivasula S.M., Ahmad M., Alnemri E.S., Wang X. (1997). Cytochrome c and datp-dependent formation of apaf-1/caspase-9 complex initiates an apoptotic protease cascade. Cell.

[107] Kominami K., Nakabayashi J., Nagai T., Tsujimura Y., Chiba K., Kimura H., Miyawaki A., Sawasaki T., Yokota H., Manabe N. (2012). The molecular mechanism of apoptosis upon caspase-8 activation: Quantitative experimental validation of a mathematical model. Biochim. Biophys. Acta.

[108] Degterev A., Huang Z., Boyce M., Li Y., Jagtap P., Mizushima N., Cuny G.D., Mitchison T.J., Moskowitz M.A., Yuan J. (2005). Chemical inhibitor of nonapoptotic cell death with therapeutic potential for ischemic brain injury. Nat. Chem. Biol..

[109] Cho Y.S., Challa S., Moquin D., Genga R., Ray T.D., Guildford M., Chan F.K. (2009). Phosphorylation-driven assembly of the rip1-rip3 complex regulates programmed necrosis and virus-induced inflammation. Cell.

[110] Zhang D.W., Shao J., Lin J., Zhang N., Lu B.J., Lin S.C., Dong M.Q., Han J. (2009). Rip3, an energy metabolism regulator that switches tnf-induced cell death from apoptosis to necrosis. Science.

[111] Scaffidi P., Misteli T., Bianchi M.E. (2002). Release of chromatin protein hmgb1 by necrotic cells triggers inflammation. Nature.

[112] Han W., Li L., Qiu S., Lu Q., Pan Q., Gu Y., Luo J., Hu X. (2007). Shikonin circumvents cancer drug resistance by induction of a necroptotic death. Mol. Cancer Ther..

[113] Jin S., White E. (2007). Role of autophagy in cancer: Management of metabolic stress. Autophagy.

[114] Komatsu M., Kominami E., Tanaka K. (2006). Autophagy and neurodegeneration. Autophagy.

[115] Mizushima N., Levine B., Cuervo A.M., Klionsky D.J. (2008). Autophagy fights disease through cellular self-digestion. Nature.

[116] Xie Z., Nair U., Klionsky D.J. (2008). Atg8 controls phagophore expansion during autophagosome formation. Mol. Biol. Cell.

[117] Nguyen T.G., Honson N.S., Arns S., Davis T.L., Dhe-Paganon S., Kovacic S., Kumar N.S., Pfeifer T.A., Young R.N. (2014). Development of fluorescent substrates and assays for the key autophagy-related cysteine protease enzyme, ATG4B. Assay Drug Dev. Technol..

[118] Martin M.D., Matrisian L.M. (2007). The other side of MMPS: Protective roles in tumor progression. Cancer Metastasis Rev..

[119] Palavalli L.H., Prickett T.D., Wunderlich J.R., Wei X., Burrell A.S., Porter-Gill P., Davis S., Wang C., Cronin J.C., Agrawal N.S. (2009). Analysis of the matrix metalloproteinase family reveals that mmp8 is often mutated in melanoma. Nat. Genet..

[120] Gutierrez-Fernandez A., Fueyo A., Folgueras A.R., Garabaya C., Pennington C.J., Pilgrim S., Edwards D.R., Holliday D.L., Jones J.L., Span P.N. (2008). Matrix metalloproteinase-8 functions as a metastasis suppressor through modulation of tumor cell adhesion and invasion. Cancer Res..

[121] Itoh Y., Seiki M. (2006). MT1-MMP: A potent modifier of pericellular microenvironment. J. Cell Physiol.

[122] Sabeh F., Ota I., Holmbeck K., Birkedal-Hansen H., Soloway P., Balbin M., Lopez-Otin C., Shapiro S., Inada M., Krane S. (2004). Tumor cell traffic through the extracellular matrix is controlled by the membrane-anchored collagenase MT1-MMP. J. Cell Biol..

[123] Lu S., Wang Y., Huang H., Pan Y., Chaney E.J., Boppart S.A., Ozer H., Strongin A.Y. (2013). Quantitative FRET imaging to visualize the invasiveness of live breast cancer cells. PLoS ONE.

[124] Yang J., Zhang Z., Lin J., Lu J., Liu B.F., Zeng S., Luo Q. (2007). Detection of mmp activity in living cells by a genetically encoded surface-displayed FRET sensor. Biochim. Biophys. Acta.

[125] Vandame P., Spriet C., Riquet F., Trinel D., Cailliau-Maggio K., Bodart J.F. (2013). Optimization of ERK activity biosensors for both ratiometric and lifetime FRET measurements. Sensors.

[126] Vandame P., Spriet C., Trinel D., Gelaude A., Caillau K., Bompard C., Biondi E., Bodart J.F. (2014). The spatio-temporal dynamics of PKA activity profile during mitosis and its correlation to chromosome segregation. Cell Cycle.

[127] Nagai T., Miyawaki A. (2004). A high-throughput method for development of FRET-based indicators for proteolysis. Biochem. Biophys. Res. Commun..

[128] Elliott A.D., Gao L., Ustione A., Bedard N., Kester R., Piston D.W., Tkaczyk T.S. (2012). Real-time hyperspectral fluorescence imaging of pancreatic beta-cell dynamics with the image mapping spectrometer. J. Cell Sci..

[129] Depry C., Zhang J. (2010). Visualization of kinase activity with FRET-based activity biosensors. Curr. Protoc. Mol. Biol..

[130] Harvey C.D., Ehrhardt A.G., Cellurale C., Zhong H., Yasuda R., Davis R.J., Svoboda K. (2008). A genetically encoded fluorescent sensor of ERK activity. Proc. Natl. Acad. Sci. USA.

[131] Aoki K., Kumagai Y., Sakurai A., Komatsu N., Fujita Y., Shionyu C., Matsuda M. (2013). Stochastic ERK activation induced by noise and cell-to-cell propagation regulates cell density-dependent proliferation. Mol. Cell.

[132] Hiratsuka T., Fujita Y., Naoki H., Aoki K., Kamioka Y., Matsuda M. (2015). Intercellular propagation of extracellular signal-regulated kinase activation revealed by *in vivo* imaging of mouse skin. Elife.

[133] Ting A.Y., Kain K.H., Klemke R.L., Tsien R.Y. (2001). Genetically encoded fluorescent reporters of protein tyrosine kinase activities in living cells. Proc. Natl. Acad. Sci. USA.

[134] Mehta S., Zhang J. (2015). Dynamic visualization of calcium-dependent signaling in cellular microdomains. Cell Calcium.

[135] Miyawaki A., Griesbeck O., Heim R., Tsien R.Y. (1999). Dynamic and quantitative Ca2+ measurements using improved cameleons. Proc. Natl. Acad. Sci. USA.

[136] Truong K., Sawano A., Mizuno H., Hama H., Tong K.I., Mal T.K., Miyawaki A., Ikura M. (2001). FRET-based *in vivo* Ca2+ imaging by a new calmodulin-gfp fusion molecule. Nat. Struct. Biol..

[137] Groffen J., Stephenson J.R., Heisterkamp N., de Klein A., Bartram C.R., Grosveld G. (1984). Philadelphia chromosomal breakpoints are clustered within a limited region, BCR, on chromosome 22. Cell.

[138] Feller S.M. (2001). CRK family adaptors-signalling complex formation and biological roles. Oncogene.

[139] Vincent A., Herman J., Schulick R., Hruban R.H., Goggins M. (2011). Pancreatic cancer. Lancet.

[140] Frame M.C. (2002). SRC in cancer: Deregulation and consequences for cell behaviour. Biochim. Biophys. Acta.

[141] Morton J.P., Karim S.A., Graham K., Timpson P., Jamieson N., Athineos D., Doyle B., McKay C., Heung M.Y., Oien K.A. (2010). Dasatinib inhibits the development of metastases in a mouse model of pancreatic ductal adenocarcinoma. Gastroenterology.

[142] Felsenfeld D.P., Schwartzberg P.L., Venegas A., Tse R., Sheetz M.P. (1999). Selective regulation of integrin—cytoskeleton interactions by the tyrosine kinase src. Nat. Cell Biol..

[143] Critchley D.R. (2000). Focal adhesions - the cytoskeletal connection. Curr. Opin. Cell Biol..

[144] Horejsi V., Zhang W., Schraven B. (2004). Transmembrane adaptor proteins: Organizers of immunoreceptor signalling. Nat. Rev. Immunol..

[145] Altan-Bonnet G., Germain R.N. (2005). Modeling t cell antigen discrimination based on feedback control of digital ERK responses. PLoS Biol..

[146] Cadra S., Gucciardi A., Valignat M.P., Theodoly O., Vacaflores A., Houtman J.C., Lellouch A.C. (2015). Roza-xl, an improved FRET based biosensor with an increased dynamic range for visualizing zeta associated protein 70 kd (zap-70) tyrosine kinase activity in live t cells. Biochem. Biophys. Res. Commun..

[147] van Beek J.D., Hess S., Vollrath F., Meier B.H. (2002). The molecular structure of spider dragline silk: Folding and orientation of the protein backbone. Proc. Natl. Acad. Sci. USA.

[148] Orr A.W., Helmke B.P., Blackman B.R., Schwartz M.A. (2006). Mechanisms of mechanotransduction. Dev. Cell.

[149] Chen C.S., Alonso J.L., Ostuni E., Whitesides G.M., Ingber D.E. (2003). Cell shape provides global control of focal adhesion assembly. Biochem. Biophys. Res. Commun..

[150] Bershadsky A.D., Balaban N.Q., Geiger B. (2003). Adhesion-dependent cell mechanosensitivity. Annu. Rev. Cell. Dev. Biol..

[151] Becker N., Oroudjev E., Mutz S., Cleveland J.P., Hansma P.K., Hayashi C.Y., Makarov D.E., Hansma H.G. (2003). Molecular nanosprings in spider capture-silk threads. Nat. Mater..

[152] Hahn C., Schwartz M.A. (2009). Mechanotransduction in vascular physiology and atherogenesis. Nat. Rev. Mol. Cell. Biol..

[153] Tzima E., Irani-Tehrani M., Kiosses W.B., Dejana E., Schultz D.A., Engelhardt B., Cao G., DeLisser H., Schwartz M.A. (2005). A mechanosensory complex that mediates the endothelial cell response to fluid shear stress. Nature.

[154] Cost A.L., Ringer P., Chrostek-Grashoff A., Grashoff C. (2015). How to measure molecular forces in cells: A guide to evaluating genetically-encoded FRET-based tension sensors. Cell. Mol. Bioeng..

[155] Drepper T., Huber R., Heck A., Circolone F., Hillmer A.K., Buchs J., Jaeger K.E. (2010). Flavin mononucleotide-based fluorescent reporter proteins outperform green fluorescent protein-like proteins as quantitative *in vivo* real-time reporters. Appl. Environ. Microbiol..

[156] White A. (1959). Effect of PH on fluorescence of tryosine, tryptophan and related compounds. Biochem. J..

